# Phytochemical Composition and Quality Attributes of Pear Cultivars Grown Under Organic and Conventional Orchard Management: A Three-Year Study

**DOI:** 10.3390/molecules31121989

**Published:** 2026-06-06

**Authors:** Ewelina Hallmann

**Affiliations:** 1 Institute of Human Nutrition Sciences, Department of Functional and Organic Food, Warsaw University of Life Sciences, Nowoursynowska 159c, 02-776 Warsaw, Poland; ewelina_hallmann@sggw.edu.pl; Tel.: +48-22-59-370-36; 2 Bioeconomy Research Institute, Agriculture Academy, Vytautas Magnus University, Donelaicio 58, 44248 Kaunas, Lithuania

**Keywords:** pears, organic, conventional, polyphenols, carotenoids, ‘Xenia’, ‘Alexander Lucas’, ‘Conference’

## Abstract

Organic fruit production is associated with a specific form of farm management: no artificial pesticides or mineral fertilizers are allowed. Only natural methods of fertilization and plant protection, including preventive practices, are used. The Organic Production Regulation describes all organic farming practices. Fruits from organic production are often perceived by consumers as safe and potentially beneficial to health. Pears contain many bioactive compounds from the polyphenol and carotenoid groups. In the present study, three pear cultivars, namely ‘Alexander Lucas’, ‘Conference’, and ‘Xenia’, grown under organic and conventional systems, were examined during the 2019–2020 cultivation season. The contents of polyphenols, carotenoids, chlorophylls, and vitamin C in pear fruits were measured using total and HPLC methods. Compared with conventional pears, organic pears were characterized by significantly higher vitamin C (8.99 mg/100 g fresh weight), total polyphenol (108.20 mg/100 g F.W.), total flavonoid (63.92 mg/100 g F.W.), total carotenoid (14.58 mg/100 g F.W.), and total chlorophyll (4.29 mg/100 g F.W.) contents. Among the three examined cultivars, ‘Xenia’ exhibited the highest concentrations of several analysed phytochemicals. The growing season significantly affected the phytochemical composition and quality attributes of pear fruits.

## 1. Introduction

Organic farming is a regulated agricultural production system based on specific management practices. The first difference between organic cultivation and other agricultural systems is the fertilization system. Artificial nitrogen fertilizers are completely forbidden in the organic fertilization system. All details concerning the use of different fertilization regimens and doses are described in rules published by the European Union [1]. A similar situation occurs with pest management. No synthetic pesticides are allowed. Only natural methods for plant protection are recommended. In organic farming systems, insect monitoring and control are commonly based on methods such as pheromone traps and yellow and white sticky traps. Preventive measures are prioritised over direct intervention strategies [2,3]. The use of natural predators, such as birds, is common in organic orchards. Moreover, this approach increases biodiversity on organic farms [4]. Pears are the most popular dessert fruit after apples in Europe. Among the European countries that are leaders in pear production, Italy ranks first, followed by Belgium, with average four-year production volumes of 378 thousand tons per year. After those countries is the Netherlands, with 278 thousand tons per year. Poland is in ninth place, with 78.9 thousand tons per year [5]. In the case of organic pears, there is no information about production volume per year. In 2024, organic pear production in Poland significantly increased, strengthening the country’s position in the European market [6]. However, detailed data specific to the organic sector for this particular year are still being processed by certification companies. The overall trend in organic farming in Poland is increasing [7]. Among pear cultivars, ‘Conference’ cv. is still the most popular, with production across the entire EU (including Poland) increasing by nearly 16%. Apart from ‘Conference’ cv., ‘Alexander Lucas’ cv., Clapp’s Favorite cv. and ‘Xenia’ cv. have a significant share of the production volume [8,9].

Pears are primarily produced for the fresh fruit market and are widely consumed as dessert fruits. In organic pears, only organic citrus pectin is permitted as a preservative. Fresh pear fruits are among the best sources of phenolic compounds. Orchard management practices may influence fruit quality and phytochemical composition. To the best of the author’s knowledge, only two experiments have compared the phytochemical composition of organically and conventionally produced pears. Organic pears have frequently been reported to contain higher concentrations of phenolic compounds than conventional pears [10,11]. Moreover, compared with fruits produced under conventional farm management, those produced under organic management also have higher phenolic compound concentrations [12,13,14]. Polyphenols are among the most extensively studied phytochemicals in fruit crops, exhibiting strong antioxidant and anticancer properties. Increased consumption of polyphenol-rich fruits has been associated with a reduced risk of several chronic diseases, including certain types of cancer, type 2 diabetes, and neurodegenerative disorders [15,16]. Many experimental studies have investigated the difference between organic and conventional fruit quality during one season. Here, a three-year experimental study with different varieties of organic pears is presented. Such results are valuable and lacking in the literature. At the same time, the selection of the studied cultivars contributes valuable insights to the current state of knowledge. It provides useful information for cultivar selection under different production systems. Therefore, the results of this study fill gaps in modern knowledge about the quality of organic and conventional pears. The hypothesis of this study was that the cultivation system, cultivar, and growing season would significantly influence the phytochemical composition and quality characteristics of pear fruits.

## 2. Results

Organic pears were characterized by significantly higher contents of vitamin C (*p* < 0.0001), total polyphenols (*p* < 0.0001), flavonoids (*p* < 0.0001), carotenoids (*p* < 0.0001) and chlorophylls (*p* < 0.0001) compared with conventional pears (Table 1). Among the three pear cultivars, ‘Xenia’ cv. exhibited the highest values for several analysed parameters. In terms of vitamin C, polyphenols and flavonoids showed the highest and most significant levels of bioactive compounds. The total carotenoid content (*p* < 0.0001) was highest in the ‘Conference’ cultivar. The total chlorophyll content was similar across all examined pears. The data presented in Table 1 show that the first year of cultivation strongly affected the concentrations of all the chemical compounds. The interactions among year, cultivation system and pear cultivar revealed the following relationships. In the case of vitamin C in 2019, in both agrosystems (organic and conventional), the highest level of vitamin C in ‘Xenia’ cv. was observed. In 2020, the highest level occurred in ‘Alexander Lucas’ cv., but no dependence was observed in the third experimental year, 2021 (Table 2).

There was no effect of pear cultivar on the dry matter content of the experimental pears. In the case of the vitamin C fraction, Lucasówka cv. and ‘Xenia’ cv. contained significantly more l-ascorbic acid (*p* < 0.0001). ‘Xenia’ cv. showed comparatively higher concentrations of dehydroascorbic acid (Table 3).

Pears from the year 2021 contained significantly more dry matter (*p* < 0.0001), but in the case of l-ascorbic acid and dehydroascorbic acid, higher contents were detected in 2019. Across the three experimental factors, the highest dry matter content in the organic system was observed in ‘Alexander Lucas’ cv. in 2019 and 2020, whereas in the conventional system it was observed in ‘Conference’ cv. in 2019 and 2021 (Table 4).

In terms of total polyphenols, ‘Xenia’ cv. during the two experimental seasons (2020 and 2021) exhibited the highest total polyphenol concentrations in both cultivation systems (organic and conventional) (Table 2). Similar results were obtained for the total flavonoid content. In 2019, only the organic system cultivar ‘Alexander Lucas’ was characterized as a cultivar with flavonoid content that was almost doubled compared with that of the other pear cultivars. The ‘Conference’ cultivar contained significantly higher levels of total carotenoids in the organic and conventional systems, but only in 2020 and 2021 (Table 2). In the first experimental year, ‘Xenia’ was the cultivar with the best total carotenoid content. In the case of the total chlorophyll content, there were no such trends when all the experimental factors were used. Only in the 2019 season did ‘Xenia’ cv. contain a significant (*p* < 0.0001) level of total chlorophyll. Moreover, in 2020 and 2021, only the organic system ‘Alexander Lucas’ cv. was characterized by a significantly higher (*p* < 0.0001) concentration of total chlorophylls.

In terms of dry matter, organic pears contained significantly more dry matter (*p* < 0.0001) compared with conventional ones. A similar situation was observed with l-ascorbic acid and dehydroascorbic acid (Table 3).

In the organic cultivation system, ‘Alexander Lucas’ cv. was characterized by a significantly higher concentration of l-ascorbic acid (*p* < 0.0001) compared with the other pear cultivars. In the conventional system, it was ‘Xenia’ cv. (Table 4). In the case of dehydroascorbic acid over the three experimental years in both systems, the cultivar ‘Xenia’ produced significantly higher levels of that compound in comparison to other examined pear cultivars. The profiling of phenolic acid and individual phenolic acid contents revealed that compared with conventional pears, organic pears were characterized by significant differences in the levels of all the identified acids (Table 5). Among the three experimental cultivars, ‘Xenia’ exhibited the highest concentrations of all identified phenolic acids except benzoic acid. Pears cultivated in the 2019 season were characterized by the highest levels of chlorogenic, *p*-coumaric and ferulic acids. Pears from 2021 contained the highest levels of gallic acid, caffeic acid and benzoic acid (Table 6). A similar situation was observed for the individual flavonoid contents. Organic pears were consistently characterized by significantly higher levels of all the identified flavonoids compared with conventional ones. Among the groups of the cultivar ‘Xenia’ cv., significantly higher catechin concentrations (*p* < 0.0001), epigallocatechin (*p* < 0.0001) and quercetin (*p* < 0.0001) were detected. All the pears from 2019 were characterized by significantly higher levels of all the identified individual flavonoids compared with those from both experimental seasons (2020 and 2021). In the organic production system, ‘Xenia’ exhibited the highest concentrations of gallic, chlorogenic, and ferulic acids across all three experimental years. In conventional orchards, such a situation was observed only with ferulic acid. However, notably, the ‘Xenia’ variety was characterized by a significantly higher content of chlorogenic acid in 2020 and 2021 and *p*-coumaric acid in 2019, 2020 and 2019. The ‘Xenia’ cultivar contained significantly more catechin and epigallocatechin in organic and conventional production systems in 2020 and 2021. The contents of myricetin and luteolin were highest in the ‘Alexander Lucas’ cultivar but were only present in organic orchards. Quercetin was a flavonoid characteristic of ‘Conference’ cv. in the conventional system in 2019 and 2021. Organic pears were characterized by significantly higher levels of lutein, zeaxanthin, beta-carotene and both chlorophyll fractions (a and b). All the identified individual carotenoid fractions showed the highest concentrations in ‘Xenia’ cv., and ‘Alexander Lucas’ cv. contained a significantly higher amount of chlorophyll a. There was no effect of cultivar on the chlorophyll b concentration. Pears that were cultivated in 2019 contained the highest levels of all the identified carotenoids, namely, lutein, zeaxanthin and beta-carotene, compared with those cultivated in the 2020 and 2021 seasons. The highest chlorophyll a concentration was observed in fruits from 2021, and the highest concentration of chlorophyll b was observed in 2019 (Table 7). In the organic cultivation system, the individual carotenoid content of Xenia cv. exhibited the highest carotenoid concentrations in 2019 and 2021. A similar situation occurred for the conventional system in all three experimental years (Table 8). In organic orchards, the cultivar ‘Alexander Lucas’ exhibited higher chlorophyll concentrations in the conventional treatment than in the cultivar ‘Xenia’, but this phenomenon was observed only in the 2019 and 2020 seasons.

**Figure 1:**
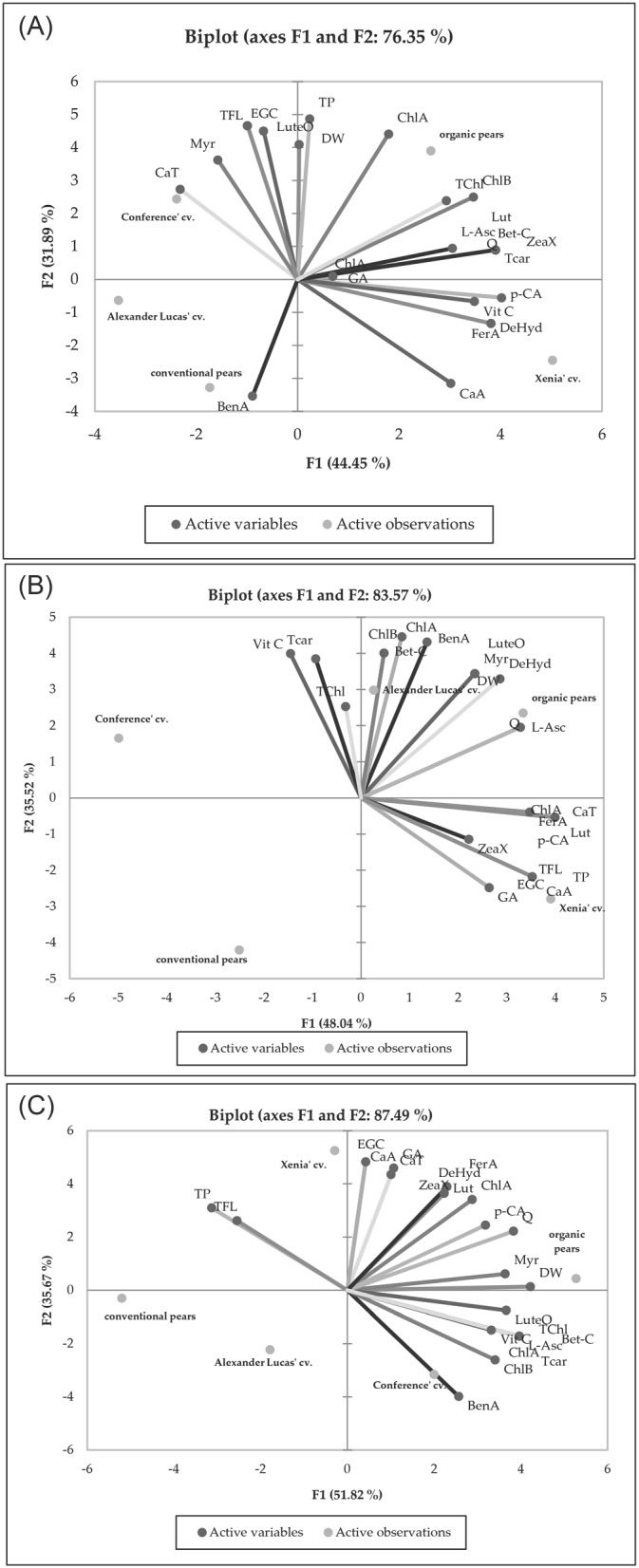
PCA showing the effects of experimental factors: farm management (organic vs. conventional) and pear cultivars, across three seasons: 2019 (**A**), 2020 (**B**), and 2021 (**C**). DW (dry matter), L-Asc (L-ascorbic acid), DeHyd (dehydroascorbic acid), GA (gallic acid), ChlA (chlorogenic acid), CaA (caffeic acid), *p*-CA (*p*-coumaric acid), FerA (ferulic acid), BenA (benzoic acid), CaT (catechin), EGC (epigallocatechin), Myr (myricetin), LuteO (luteolin), Q (quercetin), Lut (lutein), ZeaX (zeaxanthin), Bet-C (β-carotene), Chl-a (chlorophyll a), Chl-b (chlorophyll b), Vit C (vitamin C), TP (total polyphenols), TFL (total flavonoids), Tcar (total carotenoids), TChl (total chlorophylls).

Principal Component Analysis (PCA) explained 76.35% of the total variance, with PC1 and PC2 accounting for 44.45% and 31.89%, respectively. The PCA biplot revealed a clear separation between pear samples according to both farm management and cultivar. Conventional pears were located in the negative region of PC1, while organic pears were positioned on the positive side, indicating a strong influence of the cultivation system on the chemical composition. Among cultivars, ‘Xenia’ was distinctly separated along the positive PC1 and negative PC2 quadrant, whereas ‘Conference’ and ‘Alexander Lucas’ were grouped on the negative side of PC1, suggesting a similar chemical profile between these two cultivars. The distribution of variables showed that carotenoids (total carotenoids, β-carotene, lutein, zeaxanthin) and chlorophyll-related parameters (chlorophyll a, chlorophyll b and total chlorophylls) were strongly positively correlated with PC1 and associated with organic pears. In contrast, several phenolic acids (e.g., caffeic acid, ferulic acid, *p*-coumaric acid) and dehydroascorbic acid were oriented towards the lower right quadrant, contributing to the differentiation of ‘Xenia’. Flavonoids such as catechin, epigallocatechin, luteolin, and myricetin, together with total flavonoids and total polyphenols, were clustered in the upper-left quadrant, indicating mutual positive correlations and association with ‘Conference’ pears. The PCA results indicate that both cultivation system and cultivar significantly influenced the distribution of bioactive compounds in pear fruits in 2019, with clear grouping patterns driven by carotenoids, phenolic compounds, and chlorophyll content (Figure 1A).

The PCA biplot shows that the first two principal components (PC1 and PC2) explain 83.57% of the total variance, with PC1 accounting for 48.04% and PC2 for 35.52%. A clear separation of samples is observed along PC1, primarily reflecting differences between cultivation systems. Organic pears are located on the positive side of PC1, while conventional pears are positioned on the negative side, confirming a strong effect of farm management on fruit composition. Distinct cultivar-dependent clustering is also evident. ‘Xenia’ is clearly separated in the lower-right quadrant (positive PC1, negative PC2), indicating a unique chemical profile compared to the other cultivars. In contrast, ‘Conference’ is located on the negative side of PC1 and slightly positive along PC2, while ‘Alexander Lucas’ is positioned closer to the centre-right region, suggesting an intermediate profile. The distribution of variables indicates that carotenoids (total carotenoids, β-carotene, lutein, zeaxanthin) and chlorophyll-related parameters (chlorophyll a, chlorophyll b, total chlorophylls) are positively associated with PC1 and contribute to the differentiation of organic pears. Additionally, L-ascorbic acid is strongly aligned with organic samples, suggesting higher vitamin C content under organic management. Phenolic compounds, including caffeic acid, ferulic acid, *p*-coumaric acid, and total polyphenols, are oriented towards the lower-right quadrant and are strongly associated with ‘Xenia’, indicating increased accumulation of these compounds in this cultivar. In contrast, benzoic acid, luteolin, myricetin, and dehydroascorbic acid are located in the upper-right quadrant, contributing to variation along PC2. Variables grouped in similar directions (e.g., total flavonoids, total polyphenols, and caffeic acid) indicate strong positive correlations, whereas variables pointing in opposite directions reflect negative relationships. The PCA in 2020 demonstrates that both cultivation system and cultivar significantly influence the distribution of bioactive compounds, with clear separation patterns driven by carotenoids, phenolic acids, and vitamin C-related parameters (Figure 1B).

The PCA biplot indicates that the first two principal components (PC1 and PC2) explain 87.49% of the total variance, with PC1 accounting for 51.82% and PC2 for 35.67%. A strong separation of samples is observed along PC1, which primarily reflects differences between cultivation systems. Organic pears are clearly positioned on the positive side of PC1, whereas conventional pears are located on the negative side, confirming a pronounced influence of farm management on the biochemical composition of the fruit. Clear cultivar-dependent differentiation is also evident. ‘Conference’ is distinctly separated in the lower-right quadrant (positive PC1, negative PC2), while ‘Xenia’ is located in the upper-central region (slightly positive PC1, strongly positive PC2), indicating a markedly different biochemical profile. ‘Alexander Lucas’ is positioned on the negative side of PC1 and slightly negative along PC2, suggesting a profile closer to conventional samples. The distribution of variables shows that carotenoids (total carotenoids, β-carotene), chlorophylls (chlorophyll a, chlorophyll b, total chlorophyll), and vitamin C-related compounds are strongly associated with the positive side of PC1 and are closely aligned with organic pears. Dry matter and myricetin also contribute to this grouping. Phenolic acids such as caffeic acid, ferulic acid, and gallic acid, together with flavonoids (e.g., epigallocatechin), are oriented towards the upper region of the plot, contributing primarily to PC2 and distinguishing ‘Xenia’. In contrast, benzoic acid is strongly associated with ‘Conference’, as indicated by its direction towards the lower-right quadrant. Variables grouped in similar directions (e.g., total carotenoids, chlorophyll b, and β-carotene) indicate strong positive correlations, whereas those pointing in opposite directions (e.g., total polyphenols/total flavonoids vs. carotenoids) reflect negative relationships. The PCA in 2021 highlights a strong combined effect of cultivation system and cultivar, with clear separation patterns driven by pigments (carotenoids and chlorophylls), phenolic acids, and vitamin C-related compounds (Figure 1C).

## 3. Discussion

### 3.1. Polyphenol and Phenolic Acid Contents

Compared with conventional ones, consumers recognize organic fruits as better and safer. This perception is generally due to the production system and farm management, which completely excludes the use of artificial fertilizers and synthetic pesticides. Organic cultivation stimulates plants to use polyphenols as “natural” pesticides for plant protection. Organically managed apple is characterized by higher concentrations of total polyphenols and flavonoids [17]. Similar results were obtained in the present experiments with pears. Compared with conventional fruits, organic fruits contained 54.4% more total polyphenols and 39.1% more total flavonoids (Table 1). The higher content of polyphenols on the skin surface and, consequently, in pear flesh could be an effect of secondary metabolite exchange between microbiomes that colonized fruit skin and fruits. Similar findings have been reported for organic apples. Organic farming produced apples with a fruit peel microbiome enriched in plant defence, whereas conventional agriculture resulted in a microbiome enriched in pathways related to the synthesis of pesticides [18]. Apple skin polyphenols can be used to prepare apple-based antimicrobial edible films with good physical properties [19]. Some polyphenols can migrate into apple flesh and can be concentrated in a 2 mm layer under the skin. Phenolic compounds are significantly increased in fruits, with especially higher levels detected in fruit skin compared with flesh tissue, when apples from organic orchards are colonized by insects such as apple honeydew [20]. Apples produce polyphenols in their skin and fruit as signals of pest and insect attack. In the present manuscript, pears were cultivated without the spraying of any artificial pesticides on the trees. Of course, regular monitoring of insects was performed during the time of cultivation. Only natural products such as orange oil (Limoncide) and horsetail extracts were used for plant protection (Table S1, Supplementary Materials). The pear cultivar plays a significant role in insect resistance. Some cultivars, such as Mouldovia’s early or Bella di Giungo, are characterized by moderate molecular and genetic attributes and high insect resistance, containing higher levels of polyphenols compared with susceptible pear cultivars [21]. Among the three examined pear cultivars, ‘Xenia’ cv. was characterized by five of the six identified phenolic acids at dominant concentrations. Conversely, ‘Xenia’ cv. fruits contained the highest level of total polyphenols compared with the other examined pear cultivars, which was 112.68 mg/100 g F.W. The obtained results were confirmed by Gherghina et al. (2024) [22]. Among the two compared pear cultivars, ‘Alexander Lucas’ and ‘Conference’, the first was characterized by higher phenolic acid and flavonoid concentrations [23]. In the present manuscript, similar findings were obtained (Table 5). In 2019, pears were characterized by higher levels of individual polyphenols than in the other experimental years (2020 and 2021), especially in the case of chlorogenic, *p*-coumaric, and benzoic acids. Studies have indicated that phenolic compounds such as chlorogenic acid and *p*-coumaric acid are crucial for conferring this resistance.

### 3.2. Polyphenol and Flavonoid Contents

Compared with conventional pears, organic pears were characterized by a greater percentage of total flavonoids (Table 1). The concentration of flavonoids in pear skin is an effect of the skin’s reaction to UV radiation. Sunlight could be an abiotic type of stress. High radiation and temperature can affect plant function by inducing oxidative damage [24]. In response, various defence mechanisms are activated to elicit physiological, biochemical, and morphological adaptive changes [25]. The higher level of flavonoids in pear skin, especially in 2019, than in 2020 and 2021, could be due to plant reactions to sunlight and fruit exposure. According to the weather data (Figure S1, Supplementary Materials), sun exposure in the experimental orchards was much greater in 2019 than in 2020 and 2021 in the same part of the year. Notably, not only was the total level of flavonoids better in 2019, but four of the five identified individual flavonoids were also dominant in fruits from 2019 (Table 5). In the case of the luteolin and quercetin cultivar, ‘Alexander Lucas’ had the best results in organic orchards and in the 2019 season (Table 6). Similar findings were presented in other experiments with pears and apples [26]. The effect of the sun on the concentration of flavonoids during raspberry cultivation is dependent on the experimental season (2013–2014). When more sun and higher solar radiation are present, raspberries produce more flavonoids than they do during periods with less sun [26].

### 3.3. Carotenoid and Chlorophyll Contents

Pigments such as chlorophylls are responsible for the photosynthetic process and, via light absorption, produce organic compounds such as glucose. These characteristics are seen in leaf pigments. Conversely, pear fruits are mostly green in colour with a light blush on the surface. The examined pear cultivars did not produce the typical red fruit blush (Figure S1, Supplementary Materials). They remained mostly green in colour in the skin. The green pear skin contained chlorophyll pigments until the last day of harvest. After storage, the green pigments disappeared, and the surface of the pears turned yellow. The organic pears were characterized by a relatively high total chlorophyll concentration in the fruit skin (Table 1). Both the examined cultivars with fresh green skin (‘Alexander Lucas’ and ‘Xenia’ cvs. contained more total chlorophyll than ‘Conference’ did, especially in 2019 (Table 2). This difference could be an effect of higher chlorophyll activity in the sun. Sun exposure in apples provoked broad relative responses from multiple pathways, indicating a solar stress response. Responses include the accumulation of well-characterized photoprotective compounds, such as carotenoids and chlorophylls [27]. Like the flavonoid content in the three experimental seasons, the chlorophyll content and total carotenoid content in pear skin were greater in 2019, when the sun intensity and number of sunny days were much greater than those in 2020 and 2021. This phenomenon could be due to photosynthetic pigment activation via light and UV radiation (Figure S1, Supplementary Materials; Table 1 and Table 7). Chlorophyll fluorescence measurements have shown that several fruit species, such as apples and pears or mango, at least during their development and early ripening, possess functional photochemical machinery [28]. Carotenoids are bioactive compounds that protect chlorophylls against photodestruction and photooxidation [29]. Because organic pears presented higher chlorophyll concentrations, the level of carotenoids was better for protecting the whole pool of chlorophylls (Table 1 and Table 7). Similar findings have been obtained in other experiments with leafy vegetables [30].

### 3.4. The Vitamin C Content

Vitamin C is an important antioxidant produced by plants. Fresh fruits and vegetables are among the best sources of vitamin C in the diet. Fruits such as apples and pears contain moderate levels of vitamin C, but because of their high consumption frequency, they regularly deliver vitamin C to the body. According to the literature, pears contain less than 6.55–8.20 mg/100 F.W. [31]. In the present study, organic pears (8.99 mg/100 g F.W.) were characterized by a higher vitamin C concentration than conventional pears (7.44 mg/100 g F.W.). There were two cultivars, ‘Alexander Lucas’ cv. and ‘Xenia’ cv., with high concentrations of vitamin C in the fruit (Table 1). A similar relationship was presented in other experiments with pears. ‘Alexander Lucas’ cv. contained 2.51 mg/100 g F.W. of vitamin C, and ‘Xenia’ cv. contained only 2.04 mg/100 g F.W. of vitamin C. Pears synthesized more vitamin C in the skin than other fruits did in response to solar radiation. In the group of four examined cultivars, the average difference in vitamin C concentration between the skin and the pulp was 29.13% [32]. Two fractions of vitamin C, L-ascorbic acid and dehydroascorbic acid, were also predominant in organic pears (Table 3). In the year with the highest sun radiation (2019), pears were characterized by much higher levels of both vitamin C fractions. A similar situation was observed for raspberries. Compared with plants cultivated in autumn, those cultivated in summer produced more vitamin C. These phenomena were connected with the higher solar radiation in summer [14]. For consumers, fruits, even those with moderate vitamin C concentrations, constitute one of the best methods for the creation of pro-healthy behaviour. Vitamin C has strong antioxidant properties. This finding indicates that vitamin C can neutralize the negative effects of oxidative stress [33].

### 3.5. Dry Matter Content

Many experiments on organic crop quality have shown that compared with conventional fruits and vegetables, organic fruits and vegetables produce more dry matter. The results of scientific papers mostly confirm the trend towards higher contents of dry matter in plants from organic farming, especially fruits [34,35]. This phenomenon is most likely related to the type and dose of fertilizers used in cultivation. This relationship was also confirmed by the authors of an extensive literature review in which the high-dose mineral fertilization often used in conventional agriculture was linked to excessive vegetative growth and a reduction in dry matter content in agricultural crops [36,37].

## 4. Materials and Methods

### 4.1. Plant Materials

**Figure 2:**
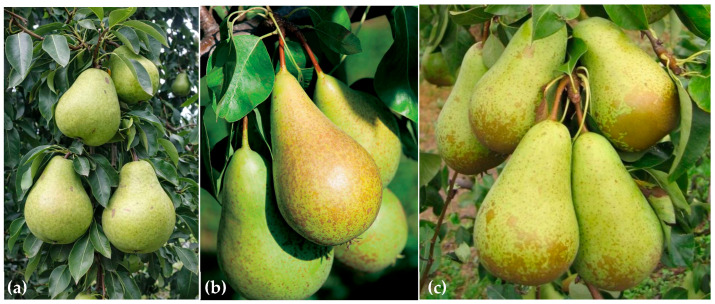
The experimental objects: (**a**) Alexander Lucas cv. (**b**) Conference cv. (**c**) Xenia cv.

**Figure 3:**
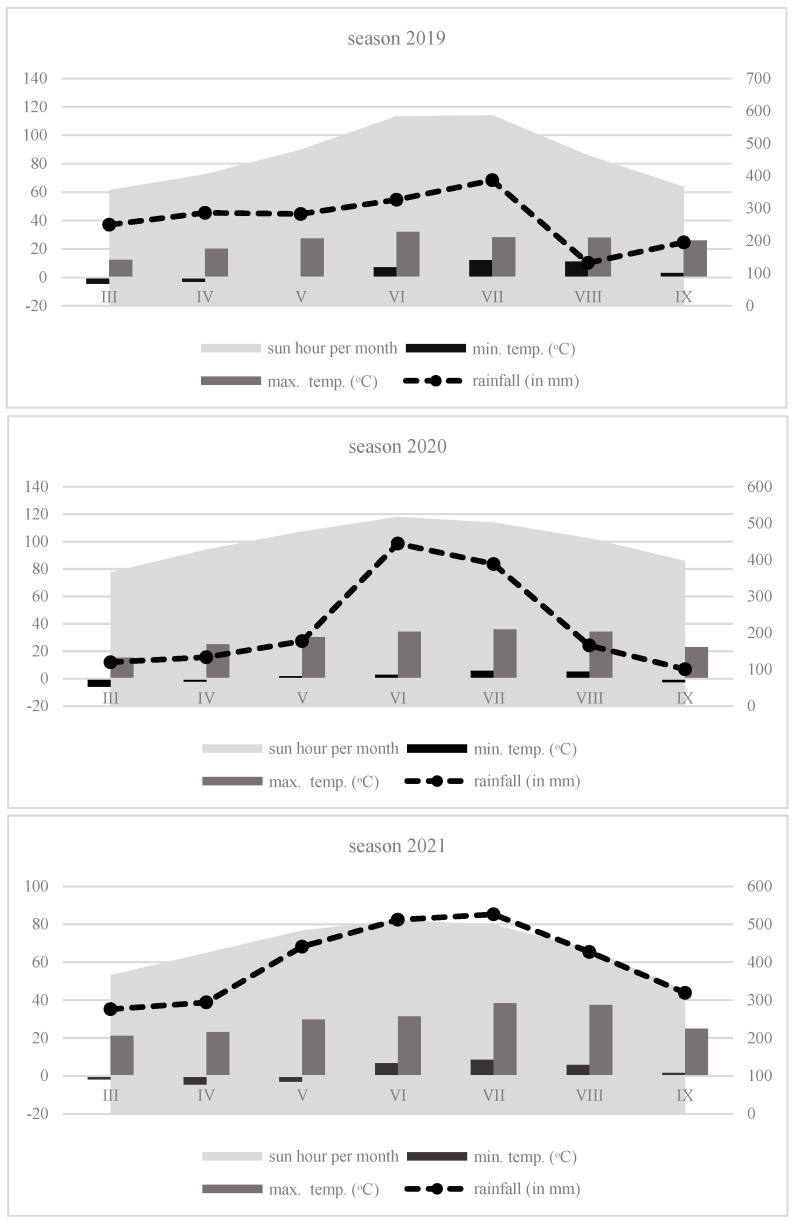
Weather conditions during three vegetable growing seasons (2019–2020 experiment).

Three pear cultivars, ‘Alexander Lucas’, ‘Conference’, and ‘Xenia’ (Figure 2), were grown across three cultivation seasons (2019, 2020, and 2021) in a certified organic orchard located in Marchaty (Łódź Voivodeship, Opoczno County, Białaczów Municipality; latitude: 51°26′28″ N, longitude: 20°19′05″ E) and a conventional orchard located in Stara Wieś (Łódź Voivodeship, Rawa County, Biała Rawska Municipality; latitude: 51°46′50″ N, longitude: 20°30′39″ E). The distance between the orchards is approximately 40 km. Detailed information on fertilization, plant protection and soil condition in production orchards is presented in Table S1 (Supplementary Materials). Weather forecasts for the three vegetable seasons during the experiment are presented in Figure 3. The experimental design schedule picture is presented in Figure S1 (Supplementary Materials). The analysis was carried out at Warsaw University of Life Sciences (WULS, Poland). Three pear cultivars consisting of a volume of 12 kg from each cultivar (plot replication: 1 kg per tree) were harvested in three replicates and delivered to the laboratory of Warsaw University of Life Sciences. From 12 trees in the orchard, 1 kg of fruit was collected. The fruit was sampled from three different parts of the tree canopy (top–middle–bottom). The fruits of the studied pear cultivars were harvested at the commercial maturity stage. One of the criteria used to assess harvest maturity was the total soluble solids (TSS) content, measured in °Brix. The average TSS content, irrespective of the production system and year of study, was 13.2 °Brix for the ‘Conference’ cultivar, 12.8 °Brix for ‘Alexander Lucas’, and 11.5 °Brix for ‘Xenia’. An additional criterion used to determine the harvest date was the number of days after full bloom (DAFB). The average harvest date occurred 141 days after full bloom for the ‘Conference’ cultivar, 149 days for ‘Alexander Lucas’, and 151 days for ‘Xenia’. Each fruit was divided into four quarters. From each fruit, one representative quarter was taken for freeze-drying, and another for dry matter determination, for a given cultivar and experimental treatment. Each sample was freeze-dried and analysed separately. The number of replicates was *n* = 36. Fresh subsamples from each sample were used to determine the dry weight. The remaining material was freeze-dried using a FreeZone™ 2.5 L Benchtop Freeze Dryer (Labconco Corporation, Kansas City, MO, USA−45 °C, 0.11 mBar), milled in an A-11 lab grinder and stored at −80 °C prior to analysis.

### 4.2. Chemicals and Reagents

Acetone (HPLC grade) was from Sigma–Aldrich (Warsaw, Poland); acetonitrile (HPLC grade) was from Sigma–Aldrich (Warsaw, Poland); aluminium chloride (ChemPur, Piekary Śląskie, Poland) and ethyl acetate (HPLC grade) were from Merck (Warsaw, Poland); Folin–Ciocâlteu reagent was from Merck (Warsaw, Poland); methanol (HPLC grade) was from Sigma–Aldrich (Warsaw, Poland); carotenoid standards (HPLC grade 99.5–99.9% pure) were from Sigma–Aldrich (Warsaw, Poland); beta-carotene, lutein, zeaxanthin, chlorophyll, chlorophyll b and magnesium carbonate (pure for analysis grade) were from ChemPur (Piekary Śląskie, Poland); oxalic acid (pure for analysis from ChemPur, Piekary Śląskie, Poland), 2,6-dichlorophenylindophenol (Sigma-Aldrich, Warsaw, Poland), gallic, chlorogenic, caffeic, *p*-coumaric, ferulic, and benzoic phenolic acid standards, and the flavonoids catechin, epigallocatechin, myritecin, luteolin, and quercetin (purity 99.0–99.9%) were from Sigma–Aldrich (Warsaw, Poland); and L-ascorbic acid (L-Asc) and dehydroascorbic acid (DHA) standards with 99% purity, orto-phosphoric acid (95% pure grade) from ChemPur, sodium acetate, and sodium carbonate were from Sigma–Aldrich (Warsaw, Poland).

### 4.3. Dry Matter Analysis Protocol

The dry matter of the examined pears was determined through the gravimetric method [38]. The empty glass beaker was weighed, and the next small portion (2 g) of catted fresh pear (flesh and skin) was poured inside. The full beaker was weighed again and placed in the laboratory dryer Farma Play FP-25 W (Bielsko-Biała, Poland). After 24 h at 105 °C, the samples were cooled in a desiccator to room temperature and weighed again. The dry matter content was calculated on the basis of differences in fresh and dry mass before and after the drying process. The results are presented as the dry matter content per g/100 g F.W.

### 4.4. Total Polyphenol Analysis Protocol

**Figure 4:**
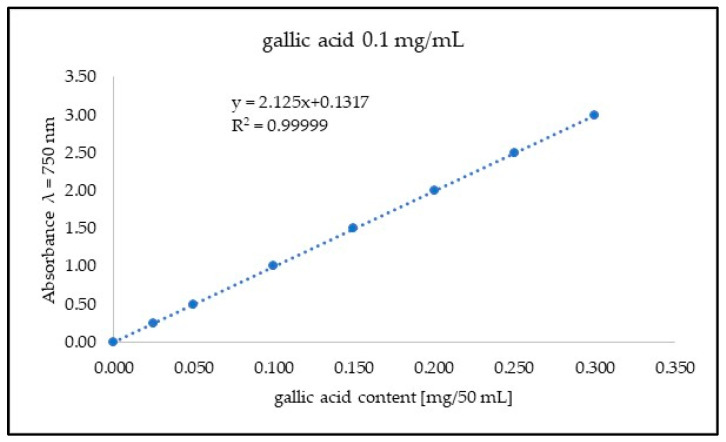
Standard curve for gallic acid (Folin–Ciocâlteu method).

The total polyphenol content was measured using the Folin–Ciocâlteu method [39]. Freeze-dried plant material (100 mg) was weighed into a 200 mL beaker, and 50 mL of 80% methanol was added. The samples were sonicated (20 min, 6000 Hz, temp. 30 °C) in an ultrasonic bath (Polsonic 9, Warsaw, Poland). The samples were then vacuum-filtered, and the obtained supernatant was used for assays. An aliquot (1.0 M) of the extract solution was transferred to a 50 mL volumetric flask; 2.5 mL of Folin–Ciocâlteu reagent and 5.0 mL of 20% sodium carbonate (Na_2_CO_3_) were added, and the volume was filled up to the mark with distilled water. The samples were incubated for 45 min at room temperature in the dark. Afterwards, the absorbance was measured at 750 nm. The total polyphenol content was calculated using a mathematical formula with the dilution factor y = (2.125 × (absorbance) + 0.1317) × 100. The results are presented as gallic acid equivalents: GAE mg/100 g F.W. (Figure 4).

### 4.5. Total Flavonoid Analysis Protocol

**Figure 5:**
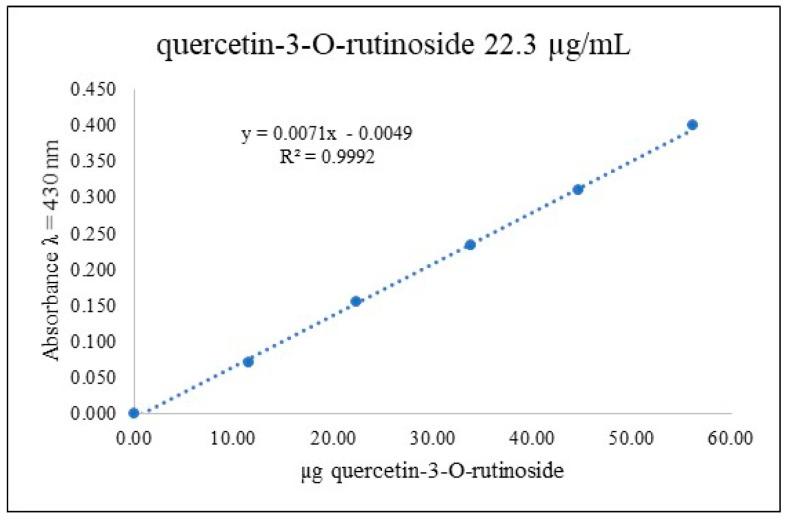
Standard curve for quercetin-3-O-rutinoside (total flavonoids method).

The total flavonoid content was measured by the colorimetric method [40]. A 100 mg freeze-dried sample was dissolved in 5 mL of 80% methanol. The sample was shaken (Micro Shaker) and incubated in an ultrasonic bath (Polsonic 9, Warsaw, Poland). Afterwards, the samples were centrifuged (6000 rpm, 10 min, temp. 5 °C). For analysis purposes, 3 mL of the extract was taken and sequentially added to 2.5 mL of sodium acetate (10%) and 1.5 mL of aluminium chloride (2.5%). Volumetric flasks were filled with 25 mL of 100% methanol and mixed. The absorbance was measured at 430 nm. A rutin (quercetin-3-O-rutinoside) calibration curve was constructed, and the results are expressed as mg/100 g F.W. (Figure 5).

### 4.6. Separation and Quantification of Individual Polyphenols Using an HPLC Protocol

Individual phenolic compounds were extracted and quantified by HPLC [41]. A 100 mg portion of powdered plant material was sonicated with 80% methanol (10 min, 6000 Hz, 30 °C). After extraction, the samples were centrifuged (10 min, 6000 rpm, 0 °C). A 50 µL aliquot of the supernatant was injected onto a Fusion RP-80 A column (250 × 4.6 mm; Phenomenex, Warsaw, Poland). Separation was performed at 1 mL min^−1^ using a gradient of acetonitrile and water acidified with phosphoric acid (pH 3.0). The pump pressure ranged from 13.00 to 14.50 mPa. The gradient programme was as follows: 1.00–24.99 min, A 95%/B 5%; 25.00–29.99 min, A 50%/B 50%; 30.00–32.99 min, A 80%/B 20%; 33.00–42.00 min, A 95%/B 5%. The total run time was 42 min. The detection wavelengths were 360 nm for flavonoids and 250 nm for phenolic acids. The compounds were identified by matching their retention times to those of external standards (gallic, chlorogenic, caffeic, *p*-coumaric, ferulic, and benzoic acids; catechin; epigallocatechin; myricetin; luteolin; quercetin). Representative chromatograms are shown in Figure S2 (Supplementary Materials). Calibration curves with equations and R^2^ values are shown in Figures S3 and S4 (Supplementary Materials). HPLC analytical parameters for standard phenolic compounds are provided in Table S2 (Supplementary Materials).

### 4.7. Protocol to Determine Total Chlorophyll and Carotenoid Contents

Total chlorophyll and carotenoid contents were determined using a colorimetric procedure [42]. A 100 mg portion of the freeze-dried plant material was placed in a glass beaker, 50 mL of cold acetone (−20 °C) was added, and the sample was thoroughly macerated using a glass rod. The mixture was transferred to a Schott funnel and vacuum-filtered through paper until the filtrate became colourless (additional cold acetone was poured until the drops ran clear). The filtrate was quantitatively transferred to a 50 mL volumetric flask, and if necessary, the volume was adjusted to the mark with acetone. The solution was mixed thoroughly, and its absorbance was recorded at 441 nm, 646 nm, and 663 nm (the spectrophotometer was calibrated with cold 80% acetone). Extinction coefficients for chlorophyll a and chlorophyll b were applied to calculate the chlorophyll a, chlorophyll b, and total carotenoid contents using the following formulas:

Chlorophyll a coefficient: (12.21 × A663) − (2.81 × A646)

Chlorophyll b coefficient: (20.13 × A646) − (5.03 × A663)

Chlorophyll a (μg g^−1^ DW) = (coef. chl. a × 50 × 1 g)/(1000 × 0.1)

Chlorophyll b (μg g^−1^ DW) = (coef. chl. b × 50 × 1 g)/(1000 × 0.1)

Total carotenoids (μg g^−1^ DW) = (1000 × (A441 − ((3.27 × coef. chl. a) − (104 × coef. chl. b))))/229

The final step involved converting the obtained dry matter content to fresh matter, and the results are expressed as mg/100 g F.W.

### 4.8. Separation and Quantification of Individual Carotenoids and Chlorophylls Using an HPLC Protocol

Carotenoid and chlorophyll contents were quantified by HPLC [41]. A 100 mg portion of freeze-dried plant material was placed into a plastic laboratory tube with acetone and 10 mg of MgCO3. Extractions were performed in a cold ultrasonic bath (10 min, 0 °C, 5.5 kHz), after which the samples were centrifuged (6000 rpm, 10 min, 0 °C). The supernatant was collected for HPLC analysis. Carotenoid separation employed a Shimadzu HPLC system (Shimadzu, Duisburg, Germany) comprising two LC-20AD pumps, a CMB-20A controller, a SIL-20AC autosampler, an SPD-20AV visible detector with spectral identification, a CTD-20A column oven, and a Max-RP 80A column (4.6 × 250 mm). The gradient mobile phase consisted of acetonitrile:methanol (90:10) as phase A and methanol:ethyl acetate (68:32) as phase B, with a flow rate of 1.0 mL·min^−1^. Detection was performed at 450 nm. Representative chromatograms are provided in Figure S5 (Supplementary Materials). Compound identification was achieved by comparison with external standards (β-carotene, lutein, zeaxanthin, chlorophyll a, and chlorophyll b; Sigma–Aldrich, Warsaw, Poland; 99.9% purity) (Supplementary Materials, Figure S6). HPLC analytical parameters for standard carotenoids and chlorophyll compounds are presented in Table S2 (Supplementary Materials).

### 4.9. Vitamin C Analysis Protocol

The vitamin C content was measured by the titration method [43]. A 100 mg portion of freeze-dried plant material was placed in a glass beaker, and 50 mL of 2% oxalic acid was added. The mixture was stirred with a glass rod, after which 2–3 drops of isoamyl alcohol were introduced. The extract was filtered through paper into a 250 mL Erlenmeyer flask using a glass funnel. An aliquot of 10 mL of the filtrate was transferred to a 100 mL Erlenmeyer flask and titrated with 2,6-dichlorophenolindophenol (*n* = 0.000896). The total vitamin C content was calculated using the following formula:

y = ml × 0.000896 × 88 × 5000

where ml is the mean titration volume, 88 is the molar equivalent for vitamin C, and 5000 is the dilution factor.

### 4.10. Separation and Quantification of Vitamin C Elements Using an HPLC Protocol

The vitamin C content in the plant materials was determined by HPLC [44]. Analyses were performed on a Shimadzu HPLC system equipped with two LC-20AD pumps, a CMB-20A controller, a SIL-20AC autosampler and an SPD-20AV visible detector with spectral capability. A 100 mg portion of freeze-dried powder was extracted with 5% meta-phosphoric acid, vortexed, sonicated (15 min, 20 °C) and centrifuged (6000 rpm, 10 min, 0 °C). A 100-μL aliquot of the supernatant was injected onto a Phenomenex Hydro 80-A RP column (250 × 4.6 mm). The chromatographic conditions included a run time of 18 min, a mobile phase of 50 mM phosphate buffer (pH 4.4) with 0.1 mM sodium acetate, and detection at 255–260 nm. L-ascorbic acid (L-Asc) and dehydroascorbic acid (DHA) were identified by comparison with the Fluka and Sigma–Aldrich reference standards (HPLC grade, 99%). HPLC analytical parameters for L-ascorbic and dehydroascorbic acids are provided in Table S2 (Supplementary Materials).

### 4.11. Statistical Methods

Chemical data were analysed using Statgraphics Centurion 15.2.11.0 (StatPoint Technologies, Inc., Warrenton, VA, USA). The experimental factors included the production method (farm management system: organic and conventional) and three pear cultivars (‘Alexander Lucas’, ‘Conference’, and ‘Xenia’). Growing seasons (2019–2021) were not included as a factor in the statistical model and are presented separately in the tables. The number of field replicates was *n* = 12 per cultivar, with *n* = 3 laboratory replicates. A total of *n* = 36 samples per cultivar within each production system were analysed in each experimental season. Statistical analysis was performed using two-way analysis of variance (ANOVA), with production method and cultivar treated as fixed factors, including their interaction. Differences between means were evaluated using Tukey’s post hoc test at a significance level of *p* ≤ 0.05. In the tables, values are presented as means ± standard deviation (SD). Means followed by the same letters do not differ significantly. Additionally, principal component analysis (PCA) was applied to explore relationships among the analysed variables.

## 5. Conclusions

Organic cultivation can influence fruit quality. Organically produced pears were characterized by higher contents of dry matter, total polyphenols, flavonoids, carotenoids, chlorophylls, and vitamin C. Among the examined cultivars, ‘Xenia’ and ‘Alexander Lucas’ have the highest amounts of these compounds. A long-term experiment would provide more detailed information about pear quality. The results differed significantly across years, mainly because of factors such as annual weather variation. However, it is worth emphasizing that certain pear varieties are well suited to organic farming: stable development and maintenance of the quality of organic pears were observed. As a practical recommendation for pear growers, the Xenia cultivar should be considered because it has shown important health benefits when it is grown organically.

## Figures and Tables

**Table 1:** Mean contents of vitamin C, total polyphenols, total flavonoids, total carotenoids, and chlorophylls (mg/100 g F.W.) in pear cultivars under two cultivation systems over three experimental years.

Experimental Combination/Examined Compound Groups	Vitamin C	Total Polyphenols	Total Flavonoids	Total Carotenoids	Total Chlorophylls
Organic pears	8.99 ± 1.63 ^a^	108.20 ± 23.94 ^a^	63.92 ± 16.72 ^a^	14.58 ± 3.34 ^a^	4.29 ± 0.49 ^a^
Conventional pears	7.44 ± 1.23 ^b^	70.06 ± 22.34 ^b^	45.96 ± 18.37 ^b^	10.43 ± 2.99 ^b^	3.40 ± 0.46 ^b^
‘Alexander Lucas’ cv.	8.65 ± 1.29 ^a^	79.59 ± 16.56 ^b^	52.70 ± 13.92 ^b^	11.59 ± 3.18 ^b^	3.80 ± 0.70 ^a^
‘Conference’ cv.	7.79 ± 1.35 ^b^	75.12 ± 11.04 ^b^	47.97 ± 16.73 ^c^	13.32 ± 3.72 ^a^	3.85 ± 0.70 ^a^
‘Xenia’ cv.	8.21 ± 2.06 ^a^	112.68 ± 22.87 ^a^	64.16 ± 11.40 ^a^	12.60 ± 4.07 ^b^	3.88 ± 0.51 ^a^
2019 year	9.73 ± 1.34 ^a^	104.64 ± 28.80 ^a^	81.63 ± 18.07 ^a^	14.76 ± 2.76 ^a^	4.16 ± 0.39 ^a^
2020 year	6.57 ± 0.59 ^b^	75.40 ± 19.89 ^b^	38.74 ± 16.78 ^b^	8.79 ± 1.77 ^b^	3.29 ± 0.49 ^b^
2021 year	8.36 ± 1.14 ^ab^	87.36 ± 13.97 ^b^	44.46 ± 10.53 ^b^	13.96 ± 3.51 ^a^	4.08 ± 0.68 ^a^
*p*-value					
System	<0.0001	<0.0001	<0.0001	<0.0001	<0.0001
Cultivars	<0.0001	<0.0001	<0.0001	<0.0001	N.S.
Year	<0.0001	<0.0001	<0.0001	<0.0001	<0.0001

Data are presented as mean ± standard deviation (SD). Means within a column followed by different letters are significantly different according to Tukey’s test (*p* < 0.05). N.S.—not significant.

**Table 2:** Mean contents of vitamin C, total polyphenols, flavonoids, carotenoids, and chlorophylls (mg/100 g F.W.) in pear cultivars under organic and conventional systems (two-way ANOVA: system × cultivar) in three experimental years.

Experimental Combination/Examined Compound Groups		Vitamin C	Total Polyphenols	Total Flavonoids	Total Carotenoids	Total Chlorophylls
Organic pears 2019	‘Alexander Lucas’ cv.	10.38 ± 0.17 ^a^	153.41 ± 11.55 ^a^	120.75 ± 12.04 ^a^	15.31 ± 0.32 ^b^	4.57 ± 0.20 ^a^
	‘Conference’ cv.	9.41 ± 0.44 ^b^	155.98 ± 3.47 ^a^	114.85 ± 4.04 ^a^	15.05 ± 0.41 ^b^	4.16 ± 0.50 ^a^
	‘Xenia’ cv.	12.36 ± 0.01 ^a^	97.20 ± 1.46 ^b^	65.81 ± 0.17 ^bc^	19.21 ± 0.10 ^a^	4.60 ± 0.28 ^a^
Conventional pears 2019	‘Alexander Lucas’ cv.	9.34 ± 0.01 ^b^	76.96 ± 12.53 ^c^	56.73 ± 1.05 ^c^	9.42 ± 0.13 ^c^	3.68 ± 0.12 ^b^
	‘Conference’ cv.	7.53 ± 0.02 ^c^	74.91 ± 0.46 ^c^	71.67 ± 11.56 ^b^	14.56 ± 0.34 ^bc^	3.77 ± 0.21 ^b^
	‘Xenia’ cv.	9.35 ± 0.01 ^b^	69.40 ± 0.07 ^c^	59.98 ± 0.63 ^c^	15.00 ± 0.34 ^b^	4.22 ± 0.10 ^a^
Organic pears 2020	‘Alexander Lucas’ cv.	7.69 ± 0.21 ^a^	65.24 ± 0.80 ^b^	31.64 ± 0.50 ^b^	10.64 ± 0.10 ^a^	4.01 ± 0.01 ^a^
	‘Conference’ cv.	6.69 ± 0.18 ^b^	63.91 ± 0.33 ^b^	26.18 ± 0.39 ^b^	11.41 ± 0.17 ^a^	3.55 ± 0.01 ^b^
	‘Xenia’ cv.	6.55 ± 0.01 ^b^	124.86 ± 0.93 ^a^	62.14 ± 0.43 ^a^	8.59 ± 0.40 ^b^	3.62 ± 0.06 ^b^
Conventional pears 2020	‘Alexander Lucas’ cv.	6.50 ± 0.11 ^b^	55.67 ± 0.12 ^c^	32.58 ± 0.04 ^b^	6.75 ± 0.29 ^c^	2.58 ± 0.05 ^c^
	‘Conference’ cv.	6.10 ± 0.18 ^b^	37.88 ± 0.57 ^d^	18.85 ± 0.58 ^c^	6.76 ± 0.30 ^c^	2.82 ± 0.03 ^c^
	‘Xenia’ cv.	5.88 ± 0.01 ^c^	104.82 ± 3.88 ^a^	61.06 ± 0.52 ^a^	8.61 ± 0.23 ^b^	3.14 ± 0.02 ^b^
Organic pears 2021	‘Alexander Lucas’ cv.	9.57 ± 0.02 ^a^	60.52 ± 0.25 ^c^	32.68 ± 0.12 ^c^	15.84 ± 0.52 ^b^	4.63 ± 0.05 ^b^
	‘Conference’ cv.	9.77 ± 0.01 ^a^	75.92 ± 0.34 ^b^	35.46 ± 0.17 ^c^	19.52 ± 0.13 ^a^	5.19 ± 0.05 ^a^
	‘Xenia’ cv.	8.54 ± 0.01 ^b^	176.75 ± 1.34 ^a^	85.82 ± 0.13 ^a^	15.62 ± 0.50 ^b^	4.25 ± 0.01 ^b^
Conventional pears 2021	‘Alexander Lucas’ cv.	8.40 ± 0.03 ^b^	65.75 ± 0.19 ^c^	41.80 ± 0.56 ^c^	11.56 ± 0.28 ^c^	3.34 ± 0.04 ^c^
	‘Conference’ cv.	7.25 ± 0.01 ^c^	42.15 ± 0.50 ^d^	20.84 ± 0.59 ^d^	12.61 ± 0.20 ^c^	3.63 ± 0.06 ^c^
	‘Xenia’ cv.	6.60 ± 0.07 ^d^	103.05 ± 0.52 ^a^	50.17 ± 0.60 ^b^	8.61 ± 0.40 ^d^	3.46 ± 0.02 ^c^
*p*-value		<0.0001	<0.0001	<0.0001	<0.0001	0.0001

Data are presented as mean ± standard deviation (SD). Means within columns followed by different letters (in every year separately) are significantly different (*p* < 0.05, Tukey’s test).

**Table 3:** Mean contents of dry matter (g/100 g F.W.) and vitamin C fractions (mg/100 g F.W.) in pear cultivars as affected by cultivation system and cultivar, presented separately for each experimental year.

Experimental Combination/Examined Compounds	Dry Matter	l-Ascorbic Acid	Dehydroascorbic Acid
Organic pears	14.73 ± 1.93 ^a^	6.89 ± 1.27 ^a^	0.75 ± 0.20 ^a^
Conventional pears	12.63 ± 1.20 ^b^	5.76 ± 1.18 ^b^	0.60 ± 0.16 ^b^
‘Alexander Lucas’ cv.	13.98 ± 2.12 ^a^	6.77 ± 1.20 ^a^	0.65 ± 0.14 ^b^
‘Conference’ cv.	13.52 ± 2.12 ^a^	5.67 ± 1.41 ^b^	0.58 ± 0.14 ^c^
‘Xenia’ cv.	13.54 ± 1.76 ^a^	6.53 ± 1.48 ^a^	0.79 ± 0.24 ^a^
2019 year	14.21 ± 2.01 ^a^	7.55 ± 0.99 ^a^	0.87 ± 0.16 ^a^
2020 year	12.54 ± 1.60 ^b^	5.09 ± 0.81 ^c^	0.53 ± 0.10 ^c^
2021 year	14.29 ± 1.70 ^a^	6.34 ± 1.09 ^b^	0.62 ± 0.14 ^b^
*p*-value			
System	<0.0001	<0.0001	<0.0001
Cultivars	N.S.	<0.0001	<0.0001
Year	<0.0001	<0.0001	<0.0001

Data are presented as mean ± standard deviation (SD). Means within a column followed by different letters are significantly different according to Tukey’s test (*p* < 0.05). N.S.—not significant.

**Table 4:** Dry matter (g/100 g F.W.) and vitamin C fractions in pear cultivars under organic and conventional systems; system × cultivar interactions analysed separately for each experimental year.

Experimental Combination/Examined Compound Groups		Dry Matter	l-Ascorbic Acid	Dehydroascorbic Acid
Organic pears 2019	‘Alexander Lucas’ cv.	17.47 ± 0.69 ^a^	8.47 ± 0.01 ^a^	0.87 ± 0.01 ^a^
	‘Conference’ cv.	15.00 ± 0.69 ^b^	7.28 ± 0.03 ^b^	0.79 ± 0.01 ^b^
	‘Xenia’ cv.	15.06 ± 0.46 ^b^	8.87 ± 0.06 ^a^	1.18 ± 0.01 ^a^
Conventional pears 2019	‘Alexander Lucas’ cv.	11.91 ± 1.16 ^d^	7.15 ± 0.50 ^b^	0.73 ± 0.06 ^b^
	‘Conference’ cv.	13.16 ± 0.53 ^c^	5.98 ± 0.56 ^c^	0.72 ± 0.06 ^b^
	‘Xenia’ cv.	12.66 ± 0.86 ^cd^	7.52 ± 0.03 ^b^	0.94 ± 0.01 ^a^
Organic pears 2020	‘Alexander Lucas’ cv.	15.31 ± 0.31 ^a^	6.42 ± 0.20 ^a^	0.72 ± 0.02 ^a^
	‘Conference’ cv.	13.29 ± 0.16 ^b^	5.16 ± 0.03 ^b^	0.54 ± 0.01 ^b^
	‘Xenia’ cv.	11.24 ± 0.34 ^c^	5.14 ± 0.13 ^b^	0.58 ± 0.02 ^b^
Conventional pears 2020	‘Alexander Lucas’ cv.	11.47 ± 0.21 ^c^	4.70 ± 0.10 ^c^	0.48 ± 0.01 ^c^
	‘Conference’ cv.	10.69 ± 0.09 ^d^	3.74 ± 0.06 ^d^	0.39 ± 0.01 ^d^
	‘Xenia’ cv.	13.23 ± 0.26 ^b^	5.40 ± 0.12 ^b^	0.48 ± 0.01 ^c^
Organic pears 2021	‘Alexander Lucas’ cv.	14.35 ± 0.12 ^b^	7.44 ± 0.12 ^a^	0.59 ± 0.01 ^c^
	‘Conference’ cv.	14.50 ± 0.33 ^b^	5.94 ± 0.20 ^c^	0.51 ± 0.01 ^d^
	‘Xenia’ cv.	16.37 ± 0.22 ^a^	7.28 ± 0.60 ^a^	0.93 ± 0.01 ^a^
Conventional pears 2021	‘Alexander Lucas’ cv.	13.35 ± 0.15 ^c^	6.45 ± 0.62 ^b^	0.53 ± 0.01 ^c^
	‘Conference’ cv.	14.50 ± 0.20 ^b^	5.94 ± 0.08 ^c^	0.51 ± 0.01 ^d^
	‘Xenia’ cv.	12.67 ± 0.25 ^d^	4.96 ± 0.13 ^d^	0.64 ± 0.01 ^b^
*p*-value		<0.0001	<0.0001	<0.0001

Data are presented as mean ± standard deviation (SD). Means within columns followed by different letters are significantly different (*p* < 0.05, Tukey’s test).

**Table 5:** Mean contents of individual phenolic compounds (mg/100 g F.W.) in pear cultivars, presented separately for cultivation system, cultivar, and experimental year.

	Gallic Acid	Chlorogenic Acid	Caffeic Acid	*p*-Coumaric Acid	Ferulic Acid	Benzoic Acid	Catechin	Epigallocatechin	Myricetin	Luteolin	Quercetin
Organic pears	15.83 ± 3.83 ^a^	19.00 ± 2.81 ^a^	0.73 ± 0.04 ^a^	0.36 ± 0.08 ^a^	0.07 ± 0.04 ^a^	1.33 ± 0.42 ^a^	10.92 ± 2.13 ^a^	43.70 ± 10.57 ^a^	0.50 ± 0.11 ^a^	0.25 ± 0.03 ^a^	0.93 ± 0.05 ^a^
Conventional pears	12.05 ± 2.40 ^b^	10.84 ± 1.98 ^b^	0.60 ± 0.03 ^b^	0.32 ± 0.08 ^b^	0.06 ± 0.03 ^a^	0.89 ± 0.27 ^b^	7.87 ± 1.66 ^b^	28.87 ± 8.83 ^b^	0.46 ± 0.10 ^b^	0.22 ± 0.02 ^b^	0.52 ± 0.05 ^b^
‘Alexander Lucas’ cv.	7.12 ± 1.63 ^b^	13.68 ± 3.22 ^b^	0.53 ± 0.12 ^ab^	0.33 ± 0.04 ^b^	0.06 ± 0.01 ^b^	1.17 ± 0.54 ^ab^	9.09 ± 1.61 ^b^	36.15 ± 9.32 ^a^	0.48 ± 0.10 ^a^	0.24 ± 0.02 ^a^	0.45 ± 0.04 ^c^
‘Conference’ cv.	7.87 ± 1.07 ^b^	13.51 ± 2.08 ^b^	0.40 ± 0.13 ^b^	0.28 ± 0.06 ^b^	0.05 ± 0.01 ^b^	1.23 ± 0.49 ^a^	4.97 ± 1.59 ^c^	34.50 ± 9.23 ^a^	0.49 ± 0.13 ^a^	0.24 ± 0.04 ^a^	0.55 ± 0.06 ^b^
‘Xenia’ cv.	26.84 ± 9.29 ^a^	17.56 ± 5.94 ^a^	1.08 ± 0.25 ^a^	0.40 ± 0.10 ^a^	0.09 ± 0.05 ^a^	0.93 ± 0.33 ^b^	14.12 ± 2.19 ^a^	38.21 ± 1.23 ^a^	0.46 ± 0.07 ^b^	0.23 ± 0.03 ^b^	1.17 ± 0.09 ^a^
2019 year	1.15 ± 0.40 ^b^	16.36 ± 5.07 ^a^	0.63 ± 0.14 ^b^	0.41 ± 0.09 ^a^	0.09 ± 0.05 ^a^	0.45 ± 0.05 ^b^	0.47 ± 0.07 ^b^	73.03 ± 15.04 ^a^	0.60 ± 0.08 ^a^	0.25 ± 0.02 ^a^	1.78 ± 0.05 ^a^
2020 year	19.23 ± 1.99 ^a^	13.34 ± 2.22 ^b^	0.67 ± 0.21 ^ab^	0.28 ± 0.04 ^c^	0.05 ± 0.01 ^b^	1.34 ± 0.70 ^a^	13.34 ± 2.47 ^a^	16.60 ± 5.64 ^b^	0.39 ± 0.04 ^b^	0.21 ± 0.03 ^b^	0.18 ± 0.03 ^b^
2021 year	21.45 ± 5.93 ^a^	15.04 ± 3.21 ^ab^	0.70 ± 0.20 ^a^	0.32 ± 0.04 ^b^	0.05 ± 0.01 ^b^	1.54 ± 0.22 ^a^	14.37 ± 2.39 ^a^	19.22 ± 3.41 ^b^	0.45 ± 0.05 ^b^	0.25 ± 0.03 ^a^	0.21 ± 0.03 ^b^
*p*-value											
System	<0.0001	<0.0001	<0.0001	<0.0001	N.S.	<0.0001	<0.0001	<0.0001	<0.0001	<0.0001	<0.0001
Cultivars	<0.0001	<0.0001	<0.0001	<0.0001	0.0001	<0.0001	<0.0001	N.S.	<0.0001	<0.0001	<0.0001
Year	<0.0001	<0.0001	0.0014	<0.0001	<0.0001	<0.0001	<0.0001	<0.0001	<0.0001	<0.0001	<0.0001

Data are presented as mean ± standard deviation (SD). Means within a column followed by different letters are significantly different according to Tukey’s test (*p* < 0.05). N.S.—not significant.

**Table 6:** Individual phenolic compounds (mg/100 g F.W.) in pear cultivars under organic and conventional systems; system × cultivar interactions presented separately for each experimental year.

System/Experimental Year	Cultivar	Gallic Acid	Chlorogenic Acid	Caffeic Acid	*p*-Coumaric Acid	Ferulic Acid	Benzoic Acid	Catechin	Epigallocatechin	Myritecin	Luteolin	Quercetin
Organic pears 2019	‘Alexander Lucas’ cv.	1.00 ± 0.10 ^b^	19.57 ± 1.07 ^b^	0.61 ± 0.06 ^b^	0.39 ± 0.02 ^b^	0.08 ± 0.01 ^b^	0.45 ± 0.02 ^b^	0.61 ± 0.04 ^a^	119.55 ± 12.06 ^a^	0.56 ± 0.01 ^b^	0.27 ± 0.002 ^a^	1.05 ± 0.15 ^b^
	‘Conference’ cv.	1.03 ± 0.02 ^b^	26.63 ± 0.64 ^a^	0.46 ± 0.02 ^c^	0.37 ± 0.01 ^b^	0.06 ± 0.01 ^c^	0.44 ± 0.02 ^b^	0.46 ± 0.02 ^b^	109.86 ± 3.74 ^a^	0.75 ± 0.01 ^a^	0.28 ± 0.001 ^a^	0.97 ± 0.15 ^b^
	‘Xenia’ cv.	2.01 ± 0.07 ^a^	28.18 ± 0.35 ^a^	0.81 ± 0.04 ^a^	0.55 ± 0.01 ^a^	0.13 ± 0.08 ^a^	0.44 ± 0.01 ^b^	0.45 ± 0.01 ^b^	46.78 ± 1.00 ^b^	0.54 ± 0.03 ^b^	0.25 ± 0.001 ^a^	5.10 ± 0.06 ^a^
Conventional pears 2019	‘Alexander Lucas’ cv.	0.85 ± 0.06 ^c^	9.52 ± 0.55 ^c^	0.50 ± 0.02 ^c^	0.32 ± 0.02 ^b^	0.07 ± 0.01 ^b^	0.39 ± 0.01 ^c^	0.40 ± 0.03 ^b^	47.16 ± 0.78 ^b^	0.66 ± 0.02 ^a^	0.21 ± 0.001 ^b^	0.84 ± 0.10 ^c^
	‘Conference’ cv.	1.10 ± 0.17 ^b^	8.77 ± 1.61 ^c^	0.64 ± 0.03 ^b^	0.32 ± 0.02 ^b^	0.06 ± 0.01 ^c^	0.45 ± 0.01 ^b^	0.49 ± 0.02 ^b^	64.37 ± 12.54 ^b^	0.57 ± 0.02 ^b^	0.26 ± 0.001 ^ab^	1.63 ± 0.12 ^b^
	‘Xenia’ cv.	0.94 ± 0.03 ^b^	5.50 ± 1.33 ^d^	0.77 ± 0.14 ^a^	0.51 ± 0.01 ^a^	0.14 ± 0.01 ^a^	0.55 ± 0.01 ^a^	0.42 ± 0.01 ^b^	50.46 ± 0.80 ^b^	0.51 ± 0.01 ^b^	0.23 ± 0.001 ^b^	1.08 ± 0.01 ^b^
Organic pears 2020	‘Alexander Lucas’ cv.	11.71 ± 0.27 ^b^	14.56 ± 0.57 ^b^	0.69 ± 0.01 ^b^	0.31 ± 0.01 ^a^	0.06 ± 0.01 ^a^	2.44 ± 0.02 ^a^	17.83 ± 0.20 ^ab^	9.92 ± 0.18 ^b^	0.47 ± 0.01 ^a^	0.26 ± 0.001 ^a^	0.23 ± 0.01 ^a^
	‘Conference’ cv.	14.54 ± 0.14 ^b^	12.67 ± 0.26 ^c^	0.31 ± 0.01 ^c^	0.26 ± 0.01 ^b^	0.04 ± 0.01 ^c^	2.10 ± 0.07 ^a^	10.00 ± 0.01 ^b^	10.09 ± 0.17 ^b^	0.40 ± 0.01 ^b^	0.23 ± 0.001 ^b^	0.19 ± 0.01 ^ab^
	‘Xenia’ cv.	35.68 ± 1.15 ^a^	16.19 ± 0.71 ^a^	1.26 ± 0.04 ^a^	0.30 ± 0.01 ^b^	0.06 ± 0.01 ^a^	1.13 ± 0.05 ^b^	20.89 ± 0.66 ^a^	32.38 ± 1.15 ^a^	0.37 ± 0.01 ^b^	0.19 ± 0.001 ^c^	0.17 ± 0.01 ^b^
Conventional pears 2020	‘Alexander Lucas’ cv.	7.45 ± 0.48 ^d^	12.37 ± 0.11 ^c^	0.34 ± 0.01 ^c^	0.27 ± 0.01 ^b^	0.05 ± 0.01 ^b^	0.68 ± 0.03 ^c^	9.03 ± 0.28 ^b^	14.30 ± 0.48 ^b^	0.36 ± 0.01 ^b^	0.20 ± 0.001 ^b^	0.16 ± 0.01 ^c^
	‘Conference’ cv.	9.87 ± 0.11 ^c^	9.45 ± 0.07 ^d^	0.23 ± 0.01 ^d^	0.20 ± 0.01 ^c^	0.03 ± 0.01 ^c^	0.60 ± 0.01 ^c^	5.56 ± 0.10 ^c^	6.10 ± 0.09 ^c^	0.34 ± 0.01 ^b^	0.18 ± 0.001 ^c^	0.14 ± 0.01 ^c^
	‘Xenia’ cv.	36.11 ± 0.60 ^a^	14.81 ± 0.56 ^b^	1.17 ± 0.01 ^a^	0.33 ± 0.01 ^a^	0.06 ± 0.01 ^a^	1.07 ± 0.03 ^b^	16.72 ± 0.39 ^ab^	26.82 ± 0.54 ^a^	0.41 ± 0.01 ^b^	0.22 ± 0.002 ^b^	0.20 ± 0.01 ^a^
Organic pears 2021	‘Alexander Lucas’ cv.	11.97 ± 0.09 ^c^	14.62 ± 0.27 ^b^	0.59 ± 0.01 ^b^	0.35 ± 0.01 ^b^	0.05 ± 0.01 ^b^	1.72 ± 0.01 ^a^	14.19 ± 0.76 ^b^	9.31 ± 0.24 ^d^	0.44 ± 0.01 ^b^	0.24 ± 0.001 ^b^	0.23 ± 0.01 ^b^
	‘Conference’ cv.	10.34 ± 0.37 ^c^	11.76 ± 1.29 ^c^	0.36 ± 0.01 ^d^	0.27 ± 0.01 ^c^	0.05 ± 0.01 ^b^	1.91 ± 0.06 ^a^	6.65 ± 0.29 ^c^	8.27 ± 0.29 ^d^	0.45 ± 0.01 ^b^	0.25 ± 0.001 ^a^	0.20 ± 0.01 ^c^
	‘Xenia’ cv.	54.23 ± 1.20 ^a^	26.78 ± 0.45 ^a^	1.45 ± 0.04 ^a^	0.41 ± 0.01 ^a^	0.07 ± 0.01 ^a^	1.36 ± 0.05 ^b^	27.17 ± 0.02 ^a^	47.17 ± 0.59 ^a^	0.53 ± 0.01 ^a^	0.28 ± 0.001 ^a^	0.26 ± 0.01 ^a^
Conventional pears 2021	‘Alexander Lucas’ cv.	9.75 ± 0.32 ^d^	11.45 ± 0.26 ^c^	0.42 ± 0.01 ^c^	0.31 ± 0.01 ^b^	0.05 ± 0.01 ^b^	1.33 ± 0.04 ^b^	12.47 ± 0.33 ^b^	16.63 ± 0.07 ^c^	0.42 ± 0.01 ^b^	0.24 ± 0.001 ^b^	0.20 ± 0.01 ^c^
	‘Conference’ cv.	10.34 ± 0.82 ^c^	11.76 ± 0.29 ^c^	0.36 ± 0.01 ^d^	0.27 ± 0.01 ^c^	0.05 ± 0.01 ^b^	1.91 ± 0.06 ^a^	6.65 ± 0.07 ^c^	8.27 ± 0.21 ^d^	0.45 ± 0.01 ^b^	0.25 ± 0.001 ^a^	0.20 ± 0.01 ^c^
	‘Xenia’ cv.	32.07 ± 0.88 ^b^	13.88 ± 0.50 ^b^	0.99 ± 0.03 ^a^	0.29 ± 0.01 ^c^	0.06 ± 0.01 ^a^	1.03 ± 0.04 ^c^	19.08 ± 0.29 ^a^	25.68 ± 0.25 ^b^	0.42 ± 0.01 ^b^	0.22 ± 0.001 ^b^	0.20 ± 0.01 ^c^
*p*-value		<0.0001	<0.0001	<0.0001	<0.0001	<0.0001	<0.0001	<0.0001	<0.0001	<0.0001	<0.0001	<0.0001

Data are presented as mean ± standard deviation (SD). Means within columns followed by different letters are significantly different (*p* < 0.05, Tukey’s test).

**Table 7:** Mean contents of individual carotenoids and chlorophylls (mg/100 g F.W.) in pear cultivars under two cultivation systems, presented separately for three experimental years.

	Lutein	Zeaxanthin	Beta-Carotene	Chlorophyll a	Chlorophyll b
Organic pears	0.60 ± 0.26 ^a^	0.58 ± 0.28 ^a^	10.43 ± 2.01 ^a^	1.70 ± 0.44 ^a^	1.49 ± 0.29 ^a^
Conventional pears	0.42 ± 0.15 ^b^	0.43 ± 0.20 ^b^	8.16 ± 1.81 ^b^	1.37 ± 0.26 ^b^	1.18 ± 0.23 ^b^
‘Alexander Lucas’ cv.	0.47 ± 0.15 ^b^	0.34 ± 0.09 ^c^	8.78 ± 1.38 ^b^	1.60 ± 0.49 ^a^	1.33 ± 0.23 ^a^
‘Conference’ cv.	0.42 ± 0.18 ^b^	0.45 ± 0.20 ^b^	9.47 ± 2.15 ^a^	1.45 ± 0.43 ^b^	1.35 ± 0.33 ^a^
‘Xenia’ cv.	0.64 ± 0.29 ^a^	0.72 ± 0.29 ^a^	9.65 ± 2.83 ^a^	1.54 ± 0.28 ^ab^	1.32 ± 0.24 ^a^
2019 year	0.74 ± 0.25 ^a^	0.73 ± 0.29 ^a^	12.32 ± 1.44 ^a^	1.25 ± 0.19 ^c^	1.93 ± 0.25 ^a^
2020 year	0.34 ± 0.08 ^b^	0.35 ± 0.10 ^c^	6.43 ± 1.35 ^c^	1.52 ± 0.37 ^b^	0.90 ± 0.21 ^b^
2021 year	0.45 ± 0.11 ^ab^	0.43 ± 0.19 ^b^	9.14 ± 1.46 ^b^	1.82 ± 0.30 ^a^	1.17 ± 0.32 ^b^
*p*-value					
System	<0.0001	<0.0001	<0.0001	<0.0001	<0.0001
Cultivars	<0.0001	<0.0001	<0.0001	0.0053	N.S.
Year	<0.0001	<0.0001	<0.0001	<0.0001	<0.0001

Data are presented as mean ± standard deviation (SD). Means within a column followed by different letters are significantly different according to Tukey’s test (*p* < 0.05). N.S.—not significant.

**Table 8:** Mean contents of individual carotenoids and chlorophylls (mg/100 g F.W.) in pear cultivars under organic and conventional systems; cultivar × system interactions presented separately for each experimental year.

System/Experimental Year	Cultivar	Lutein	Zeaxanthin	Beta-Carotene	Chlorophyll a	Chlorophyll b
Organic pears 2019	‘Alexander Lucas’ cv.	0.75 ± 0.01 ^b^	0.51 ± 0.01 ^b^	12.63 ± 0.11 ^b^	1.14 ± 0.01 ^b^	2.25 ± 0.01 ^a^
	‘Conference’ cv.	0.75 ± 0.01 ^b^	0.80 ± 0.03 ^a^	12.40 ± 0.10 ^b^	1.33 ± 0.01 ^b^	1.89 ± 0.01 ^b^
	‘Xenia’ cv.	1.22 ± 0.01 ^a^	1.24 ± 0.02 ^a^	16.04 ± 0.01 ^a^	1.56 ± 0.01 ^a^	2.22 ± 0.01 ^a^
Conventional pears 2019	‘Alexander Lucas’ cv.	0.45 ± 0.02 ^d^	0.38 ± 0.02 ^c^	7.73 ± 0.01 ^c^	0.95 ± 0.07 ^c^	1.69 ± 0.13 ^c^
	‘Conference’ cv.	0.53 ± 0.03 ^c^	0.55 ± 0.03 ^b^	12.55 ± 0.11 ^b^	1.21 ± 0.11 ^b^	1.66 ± 0.15 ^c^
	‘Xenia’ cv.	0.75 ± 0.04 ^b^	0.89 ± 0.08 ^a^	12.57 ± 0.72 ^b^	1.32 ± 0.01 ^b^	1.85 ± 0.04 ^b^
Organic pears 2020	‘Alexander Lucas’ cv.	0.44 ± 0.01 ^a^	0.29 ± 0.01 ^b^	7.87 ± 0.19 ^b^	2.23 ± 0.04 ^a^	1.14 ± 0.02 ^a^
	‘Conference’ cv.	0.32 ± 0.02 ^b^	0.46 ± 0.01 ^a^	8.39 ± 0.09 ^a^	1.75 ± 0.03 ^b^	1.20 ± 0.01 ^a^
	‘Xenia’ cv.	0.38 ± 0.01 ^b^	0.44 ± 0.01 ^a^	6.08 ± 0.20 ^b^	1.31 ± 0.05 ^c^	0.78 ± 0.02 ^b^
Conventional pears 2020	‘Alexander Lucas’ cv.	0.28 ± 0.01 ^c^	0.23 ± 0.01 ^b^	4.97 ± 0.12 ^c^	1.33 ± 0.03 ^c^	0.65 ± 0.02 ^c^
	‘Conference’ cv.	0.21 ± 0.01 ^c^	0.22 ± 0.01 ^b^	4.79 ± 0.14 ^c^	1.10 ± 0.02 ^d^	0.73 ± 0.01 ^b^
	‘Xenia’ cv.	0.38 ± 0.01 ^b^	0.45 ± 0.01 ^a^	6.47 ± 0.01 ^b^	1.44 ± 0.03 ^c^	0.91 ± 0.03 ^ab^
Organic pears 2021	‘Alexander Lucas’ cv.	0.54 ± 0.02 ^a^	0.36 ± 0.02 ^c^	10.33 ± 0.05 ^a^	2.16 ± 0.02 ^a^	1.29 ± 0.01 ^a^
	‘Conference’ cv.	0.35 ± 0.02 ^b^	0.33 ± 0.02 ^c^	9.33 ± 0.38 ^b^	1.67 ± 0.05 ^c^	1.31 ± 0.04 ^a^
	‘Xenia’ cv.	0.62 ± 0.10 ^a^	0.79 ± 0.05 ^a^	10.81 ± 0.12 ^a^	2.13 ± 0.02 ^a^	1.30 ± 0.03 ^a^
Conventional pears 2021	‘Alexander Lucas’ cv.	0.35 ± 0.04 ^b^	0.27 ± 0.01 ^d^	9.13 ± 0.20 b^c^	1.81 ± 0.02 ^b^	0.94 ± 0.01 ^b^
	‘Conference’ cv.	0.35 ± 0.01 ^b^	0.33 ± 0.01 ^c^	9.33 ± 0.23 ^b^	1.67 ± 0.02 ^c^	1.31 ± 0.01 ^a^
	‘Xenia’ cv.	0.49 ± 0.01 ^b^	0.52 ± 0.02 ^b^	5.93 ± 0.22 ^c^	1.51 ± 0.04 ^c^	0.88 ± 0.02 ^b^
*p*-value		<0.0001	<0.0001	<0.0001	<0.0001	<0.0001

Data are presented as mean ± standard deviation (SD). Means within columns followed by different letters are significantly different (*p* < 0.05, Tukey’s test).

## Data Availability

The original contributions presented in this study are included in the article. Further inquiries can be directed to the corresponding author.

## Supplementary Materials

The following supporting information can be downloaded from https://www.mdpi.com/article/10.3390/molecules31121989/s1: Figure S1: Experimental design: layout of trees in the production orchard, indicating the trees sampled during the study period (2019–2021); Figure S2: Example chromatogram of phenolic compound separation in the conventional [A] and the organic [B] pear Xenia cv.; Figure S3: Calibration curves for identified phenolic acids in pear fruits.; Figure S4: Calibration curves for identified flavonoids in pear fruits; Figure S5: Example chromatogram of carotenoid and chlorophyll separation in the conventional [A] and the organic [B] pear Xenia cv.; Figure S6: Calibration curves for identified carotenoids and chlorophylls in pear fruits; Table S1. Fertilization and plant protection in experimental orchards during pear cultivation. Table S2. HPLC analytical parameters for the standard compounds (mg per 100 g^−1^ F.W.).
