# Supplementary material for: Phytochemical Composition and Quality Attributes of Pear Cultivars Grown Under Organic and Conventional Orchard Management: A Three-Year Study
Source: Molecules. 2026 Jun 6;31(12):1989. doi: 10.3390/molecules31121989 (PMC13306168; doi:10.3390/molecules31121989)
Supplement: Supplementary file 1 [file molecules-31-01989-s001.zip › molecules-4327712-supplementary.pdf]

Table S1. Fertilization practices, plant protection, and soil conditions in experimental pear orchards.

Cultivation type/activity	Fertilization	Plant protection	Soil conditions
Organic orchard	• Organic animal manure was applied at 30 t/ha;<br>• calcium carbonate (lime) was used for soil pH regulation;<br>• Farma EM Plus (effective microorganisms)<br>• rock phosphate (PolPower) were also applied.	• Limocide (orange oil-based) was used against pear psylla;<br>• Pheromone traps and granulosis virus-based preparations (Carpovirusine) were applied for codling moth;<br>• Viflo (copper–boron) was used against fire blight and Pseudomonas spp.;<br>• Lycos (horsetail extract) was applied for pear rust.	The soil was classified as sandy loam, with an organic matter content of 4.2%, groundwater level at 1.7 m, and pH ranging from 6.2 to 6.7.
Conventional orchard	• Ammonium nitrate was applied at 20–40 kg N/ha;<br>• Potassium sulfate at 60–80 kg/ha;<br>• Fertimax Complex at 120 kg/ha.	• Dyno 2.5 EC (pear psylla) at 0.5 L/ha;<br>• Mospilan 20 SP (codling moth) at 0.4 L/ha;<br>• Copper WP 50 (fire blight) at 0.75 kg/ha;<br>• Scorpion 325 SC (rust) at 0.1 L/ha.	The soil was classified as sandy loam, with an organic matter content of 3.9%, groundwater level at 1.9 m, and pH ranging from 6.0 to 6.5.

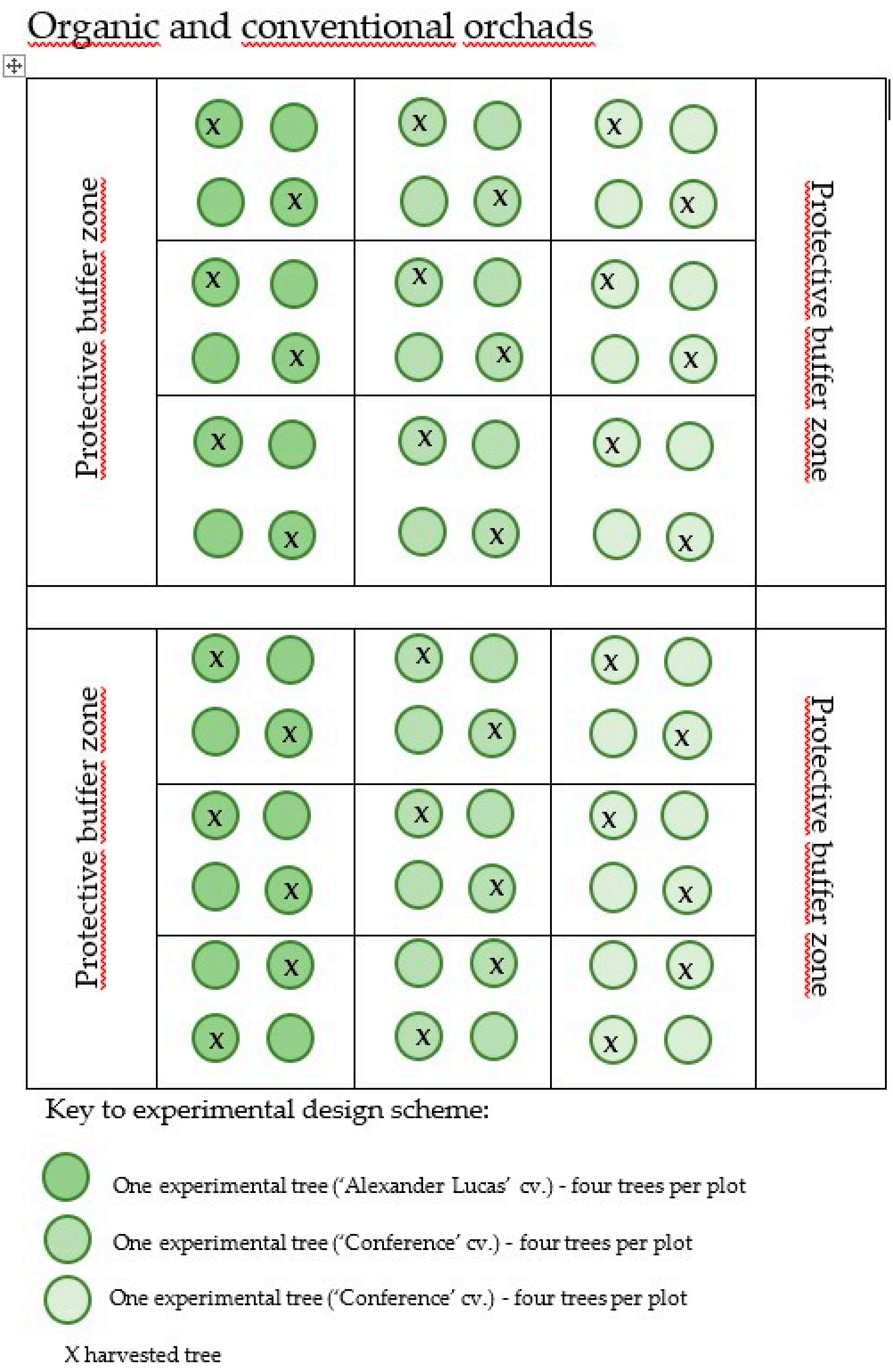

Figure S1. Experimental design: layout of trees in the production orchard, indicating the trees sampled during the study period (2019–2021).

Table S2. HPLC analytical parameters for the standard compounds (mg per 100 g^{-1} F.W.).

compounds (standard)	mg per 100 g^{-1} F.W.			
	LOD	LOQ	% recovery	% RSD for peak area
gallic acid	0.21	0.63	99.10	1.31
chlorogenic acid	1.89	5.67	97.08	1.10
caffeic acid	0.08	0.24	99.73	1.24
p-coumaric acid	0.05	0.15	98.34	1.34
ferulic acid	0.009	0.028	99.80	0.98
benzoic acid	0.120	0.360	98.90	0.92
catechin	0.12	0.37	99.92	1.26
epigallocatechin	1.81	5.44	98.88	1.39
myricetin	0.10	0.31	99.31	1.18
luteolin	0.051	0.153	99.14	1.28
quercetin	0.031	0.093	98.94	1.13
lutein	0.051	0.153	97.33	1.25
zeaxanthin	0.062	0.186	98.24	1.35
beta-carotene	1.45	4.35	99.46	1.01
chlorophyll a	0.26	0.78	99.71	2.45
chlorophyll b	0.18	0.55	97.59	2.18
l-ascorbic acid	1.20	3.62	99.73	2.21
dehydroascorbic acid	0.12	0.36	98.85	2.33

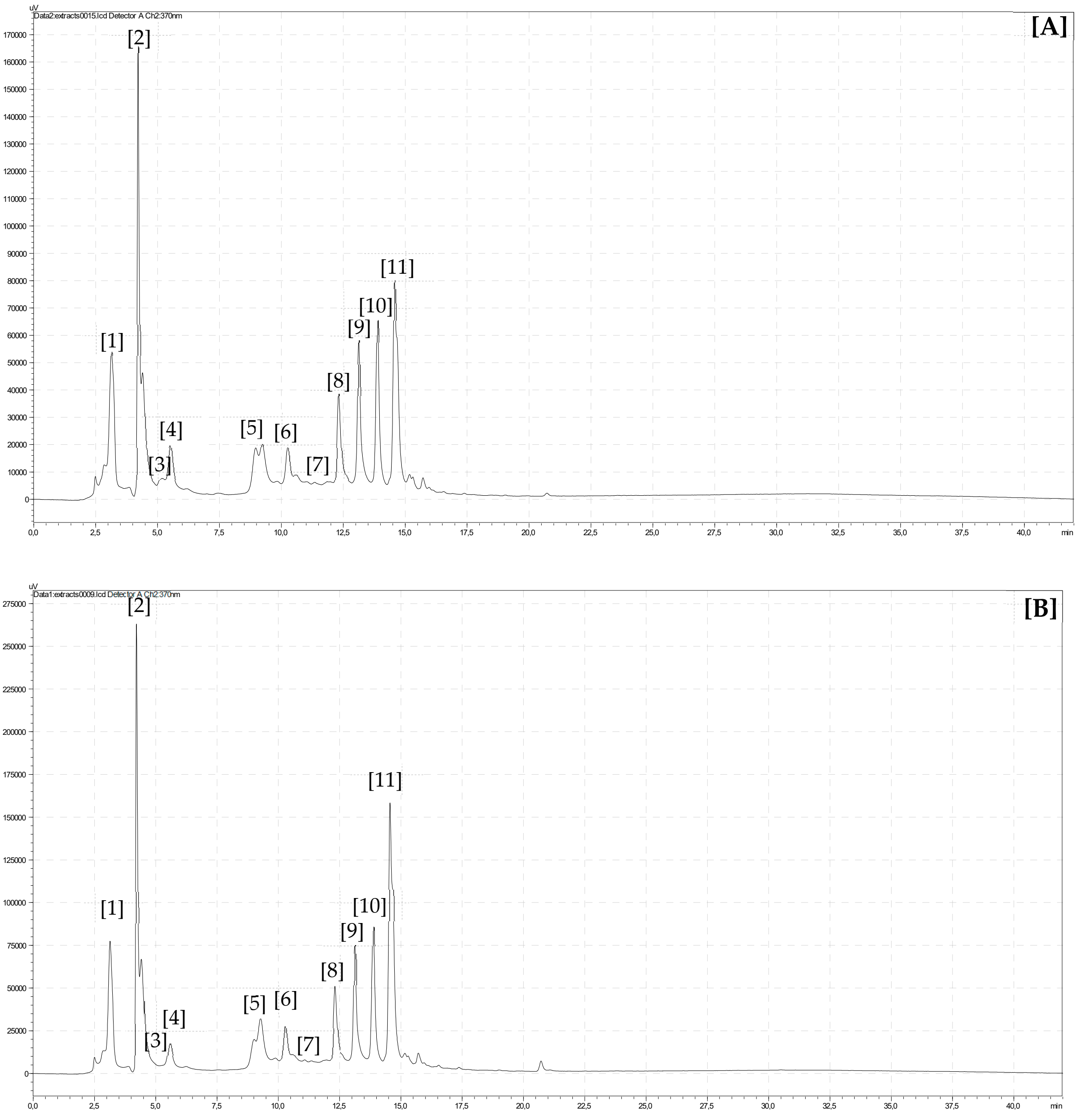

Figure S2. Representative chromatogram of phenolic compound separation in conventionally [A] and organically [B] grown *Pyrus communis* L. (Xenia cv.). Compounds: (1) gallic acid, (2) chlorogenic acid, (3) caffeic acid, (4) *p*-coumaric acid, (5) ferulic acid, (6) benzoic acid, (7) catechin, (8) epigallocatechin, (9) myricetin, (10) luteolin, (11) quercetin.

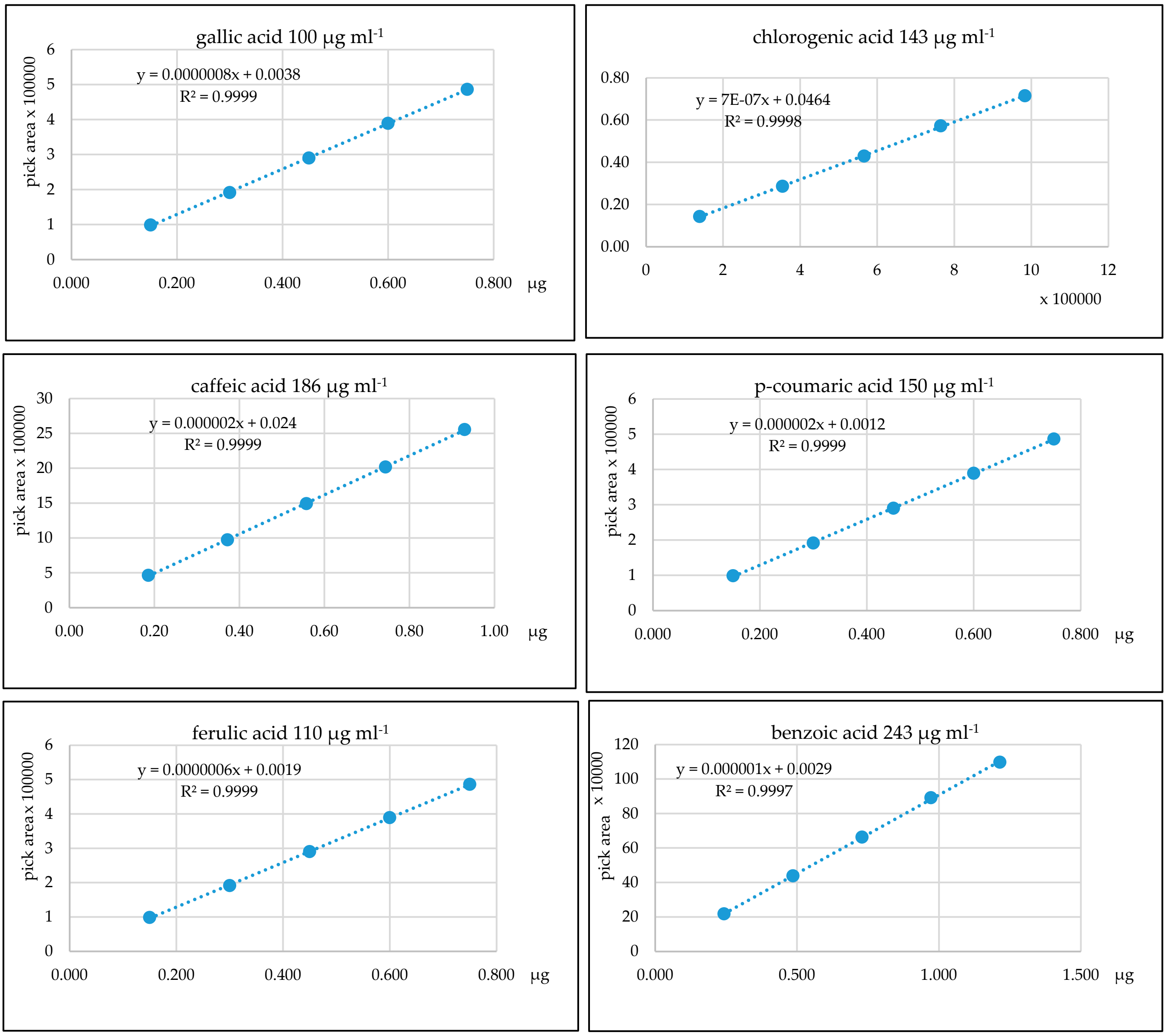

Figure S3. Calibration curves for identified phenolic acids in pear fruits.

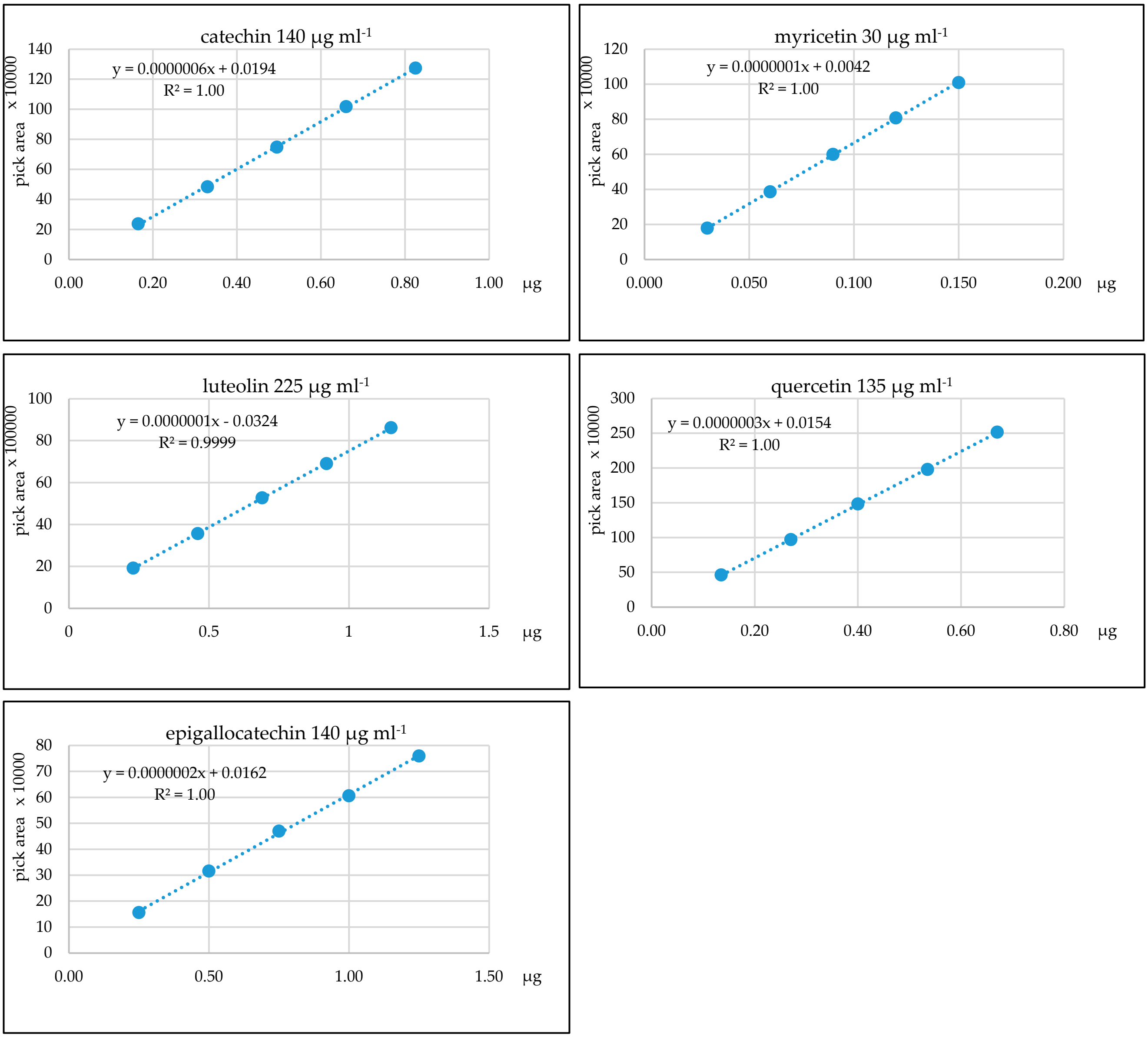

Figure S4. Calibration curves for identified flavonoids in pear fruits.

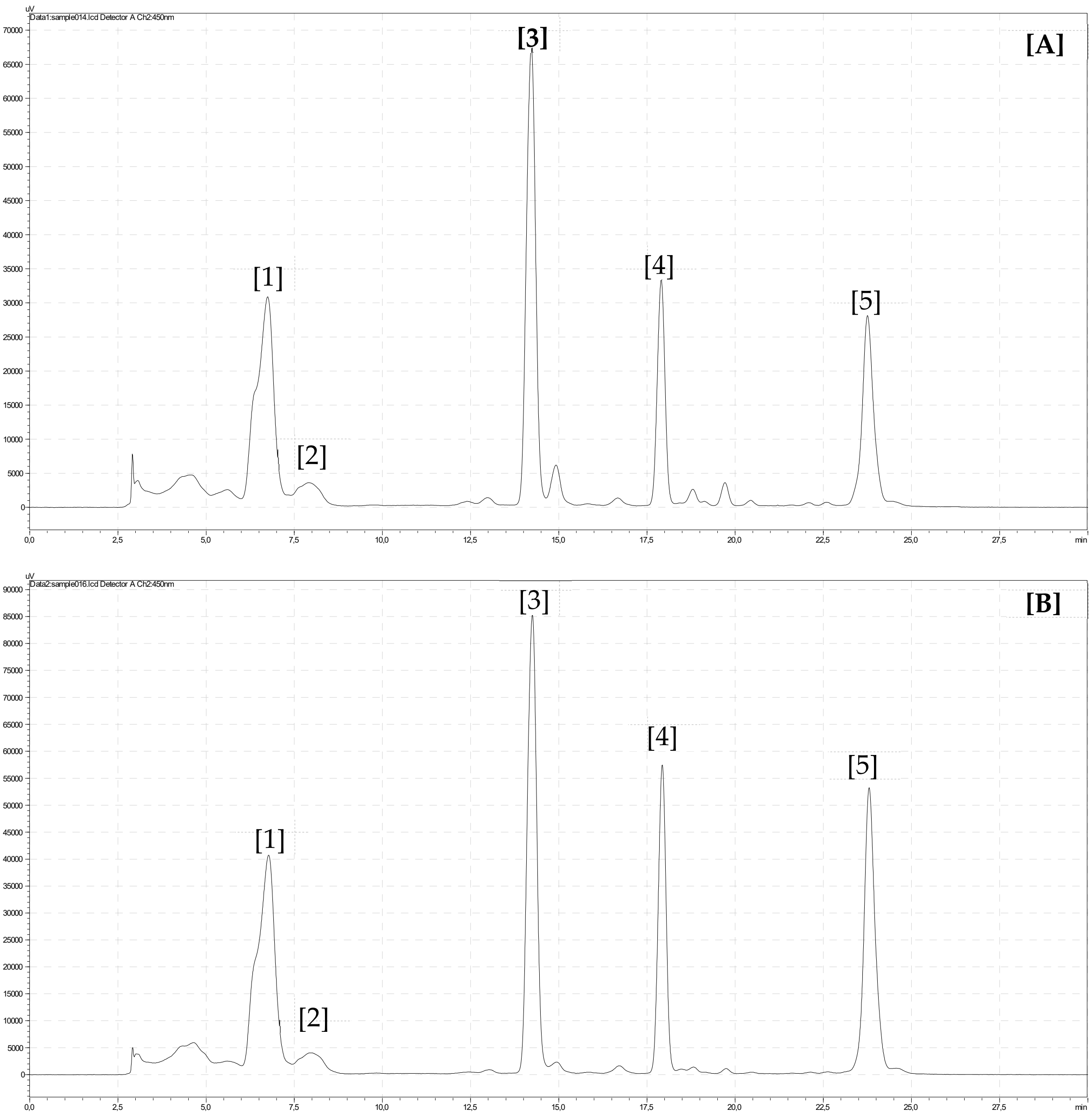

Figure S5. Representative chromatogram of carotenoid and chlorophyll separation in conventionally [A] and organically [B] grown *Pyrus communis* L. (Xenia cv.). Compounds: (1) lutein, (2) zeaxanthin, (3) chlorophyll b, (4) chlorophyll a, (5) β-carotene.

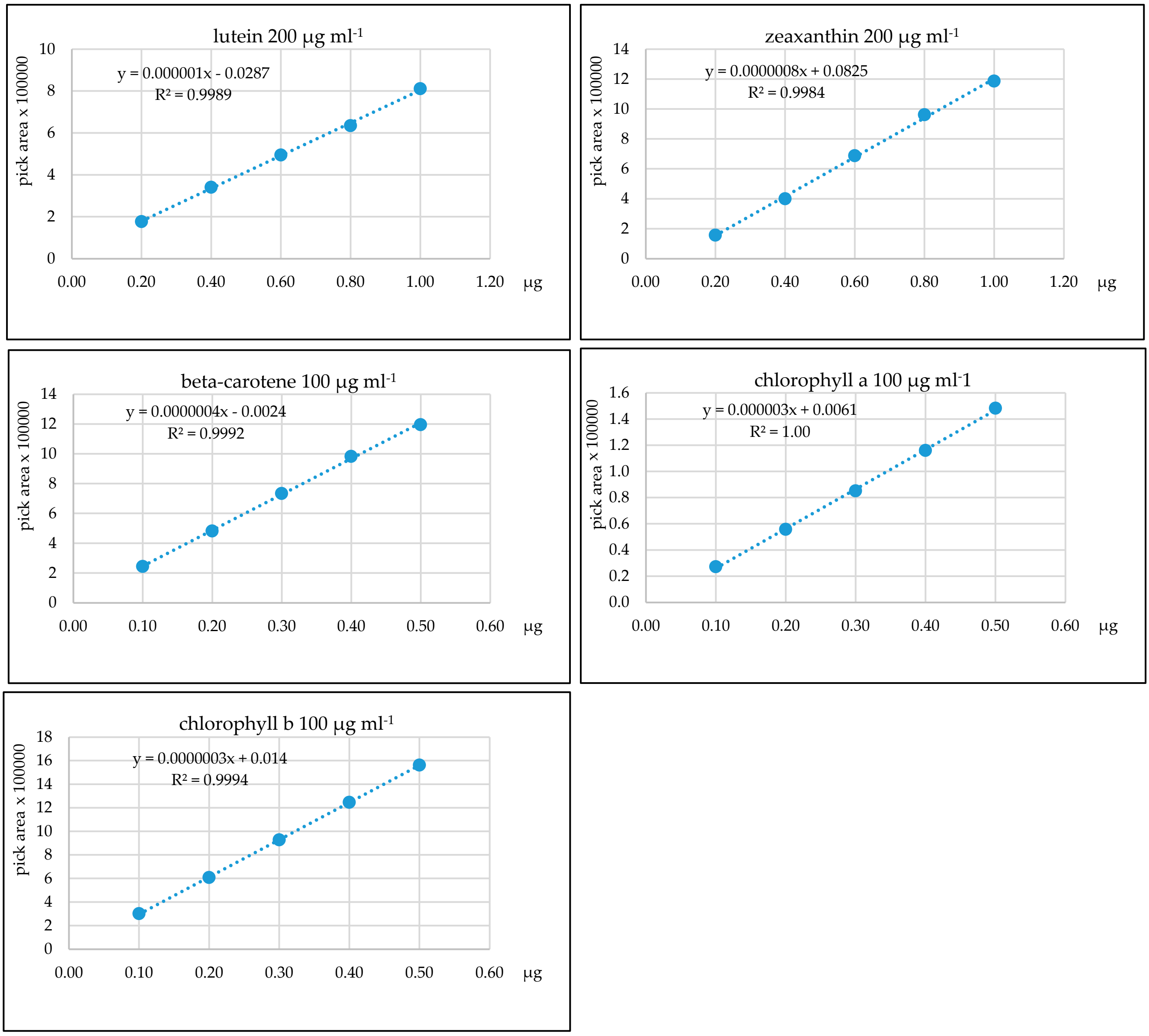

Figure S6. Calibration curves for identified carotenoids and chlorophylls in pear fruits
